# Supplementary material for: The genetic architecture of membranous nephropathy and its potential to improve non-invasive diagnosis
Source: Nat Commun. 2020 Mar 30;11:1600. doi: 10.1038/s41467-020-15383-w (PMC7105485; doi:10.1038/s41467-020-15383-w)
Supplement: Supplementary file 1 — Supplementary Information [file 41467_2020_15383_MOESM1_ESM.pdf]

SUPPLEMENTARY INFORMATION:

Genetic architecture of membranous nephropathy and its diagnostic implications

Xie et al.

Table of Contents

SUPPLEMENTARY FIGURES

Supplementary Figure 1. ... 3
Supplementary Figure 2. ... 4
Supplementary Figure 3. ... 5
Supplementary Figure 4. ... 6
Supplementary Figure 5. ... 7
Supplementary Figure 6. ... 8
Supplementary Figure 7. ... 9
Supplementary Figure 8. ... 10
Supplementary Figure 9. ... 11
Supplementary Figure 10. ... 12
Supplementary Figure 11. ... 13
Supplementary Figure 12. ... 14
Supplementary Figure 13. ... 15
Supplementary Figure 14. ... 16
Supplementary Figure 15. ... 17

SUPPLEMENTARY TABLES

Supplementary Table 1. ... 18
Supplementary Table 2. ... 19
Supplementary Table 3. ... 20
Supplementary Table 4. ... 21
Supplementary Table 5. ... 22
Supplementary Table 6. ... 23
Supplementary Table 7. ... 24
Supplementary Table 8. ... 25
Supplementary Table 9. ... 26
Supplementary Table 10. ... 27
Supplementary Table 11. ... 28
Supplementary Table 12. ... 29
Supplementary Table 13. ... 30
Supplementary Table 14. ... 31
Supplementary Table 15. ... 32
Supplementary Table 16. ... 33
Supplementary Table 17. ... 34
Supplementary Table 18. ... 35
Supplementary Table 19. ... 36
Supplementary Table 20. ... 37
Supplementary Table 21. ... 38
Supplementary Table 22. ... 39

SUPPLEMENTARY NOTES

Supplementary Note 1. ... 40
Supplementary Note 2. ... 42

SUPPLEMENTARY METHODS

Supplementary Methods ... 43

SUPPLEMENTARY REFERENCES

Supplementary References ... 53

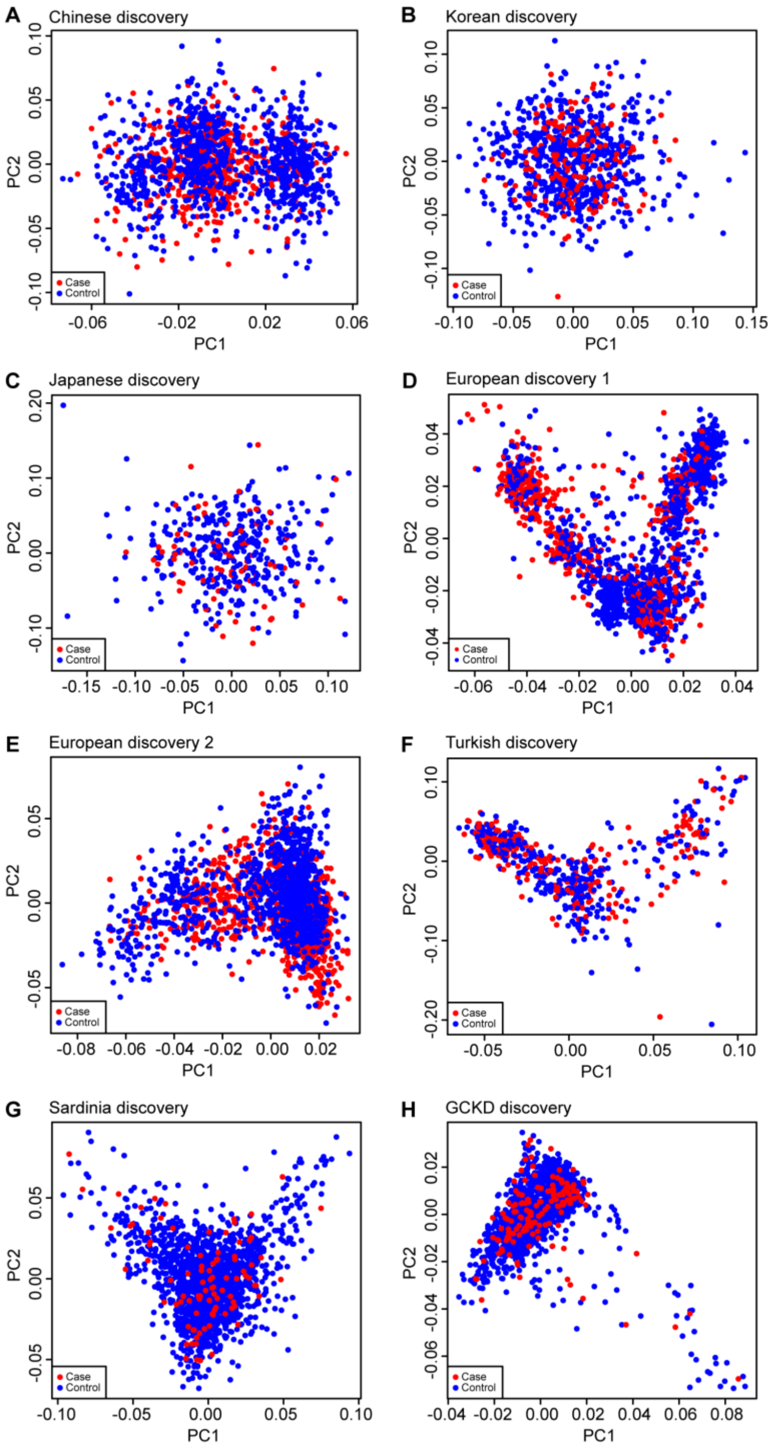

Supplementary Figure 1. Principal component plots for discovery cohorts by case-control status: (A) Chinese discovery cohort of 561 cases (red) and 904 controls (blue); **(B)** Korean discovery cohort of 164 cases and 708 controls; **(C)** Japanese discovery cohort of 81 cases and 358 controls; **(D)** European discovery-1 cohort of 611 cases and 1,246 controls; **(E)** European discovery-2 cohort of 1,045 cases and 1,094 controls; **(F)** Turkish discovery cohort of 254 cases and 336 controls; **(G)** Sardinia discovery cohort of 93 cases and 1,498 controls; and **(H)** the GCKD discovery cohort of 147 cases and 1,655 controls.

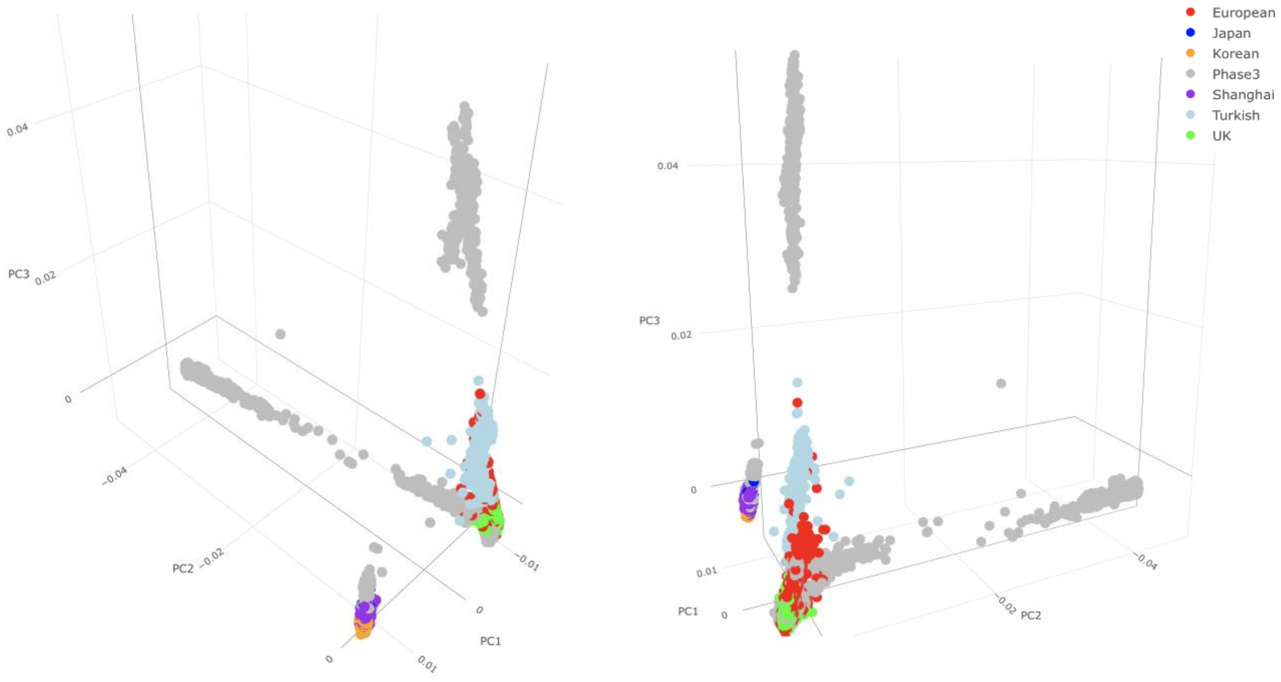

Supplementary Figure 2. Principal component plots for the discovery cohorts in relationship to 1000 Genomes Phase III reference populations. The reference populations are shaded in gray and the study case-control cohorts are depicted in colors (red: European, blue: Japanese, orange: Korean, purple: Chinese, light blue: Turkish, and green: UK). The first three principal components (PC1-3) are depicted and two views of the three-dimensional PC space are displayed to better highlight the observed separation between East Asian and European cohorts. Overall, we observe the expected mapping of our cohorts to the European and East Asian reference clusters: our Turkish, UK, and other European case-control cohorts co-cluster with the phase III Europeans, while the Japanese, Chinese, and South Korean case-control cohorts co-cluster closely with phase III East Asian populations.

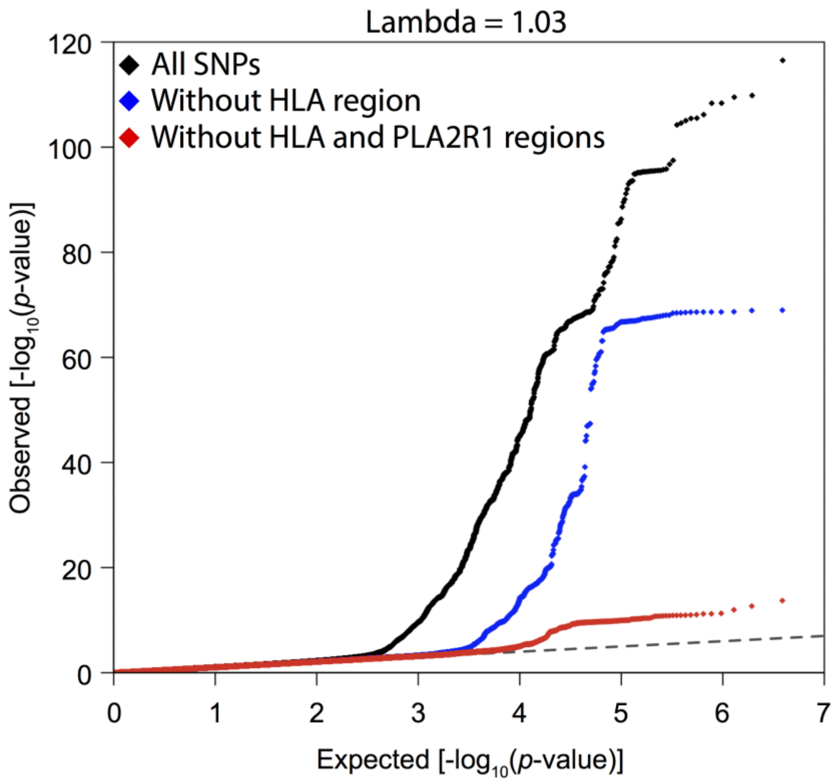

Supplementary Figure 3. Quantile-quantile (QQ) plot for the discovery meta-analysis. The black points represent QQ-plot based on all SNPs; the blue points represent the QQ plot after excluding HLA region; the red points represent QQ-plot after excluding both HLA and PLA2R1 regions. The overall genome inflation factor (lambda) was estimated at 1.03.

A *PLA2R1* locus

rs17831251	Odds ratio	Lower.limit	Upper.limit	P value
Chinese discovery cohort	3.04	2.45	3.78	1.2E-23
Korean discovery cohort	2.18	1.58	3.02	2.5E-06
Japanese discovery cohort	1.15	0.81	1.62	0.44
Turkish discovery cohort	1.94	1.43	2.62	1.7E-05
European discovery cohort 1	2	1.67	2.39	4.1E-14
European discovery cohort 2	2.11	1.84	2.41	5.4E-27
Sardinia discovery cohort	1.16	0.84	1.62	0.36
GCKD discovery cohort	2.24	1.69	2.97	2.8E-09
Chinese replication cohort	3.79	3.26	4.39	1.2E-42
**All combined**	**2.25**	**2.09**	**2.42**	**4.7E-103**

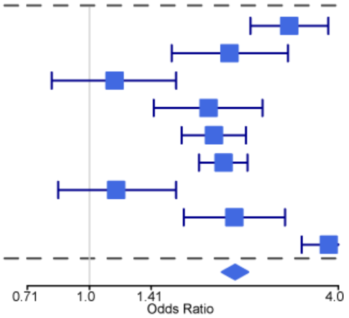

B *NFKB1* locus

rs230540	Odds ratio	Lower.limit	Upper.limit	P value
Chinese discovery cohort	1.22	1.04	1.42	0.012
Korean discovery cohort	1.53	1.19	1.97	0.00087
Japanese discovery cohort	1.66	1.17	2.34	0.0042
Turkish discovery cohort	1.66	1.26	2.18	0.00031
European discovery cohort 1	1.18	1	1.39	0.056
European discovery cohort 2	1.14	1	1.31	0.05
Sardinia discovery cohort	1.66	1.22	2.24	0.0013
GCKD discovery cohort	1.3	1.01	1.67	0.046
Chinese replication cohort	1.15	1.01	1.31	0.042
**All combined**	**1.25**	**1.17**	**1.33**	**3.4E-12**

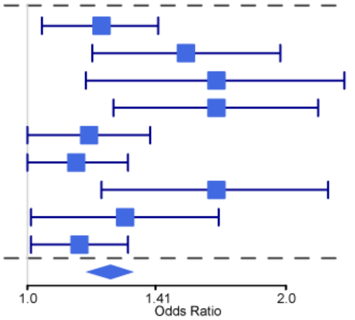

C *IRF4* locus

rs9405192	Odds ratio	Lower.limit	Upper.limit	P value
Chinese discovery cohort	1.51	1.29	1.77	2.4E-07
Korean discovery cohort	1.6	1.25	2.05	0.00018
Japanese discovery cohort	1.1	0.75	1.62	0.62
Turkish discovery cohort	1.1	0.84	1.42	0.49
European discovery cohort 1	1.26	1.06	1.49	0.0075
European discovery cohort 2	1.2	1.03	1.4	0.019
Sardinia discovery cohort	1.28	0.92	1.79	0.13
GCKD discovery cohort	0.95	0.73	1.23	0.71
Chinese replication cohort	1.32	1.17	1.49	1.2E-05
**All combined**	**1.29**	**1.21**	**1.37**	**1.4E-14**

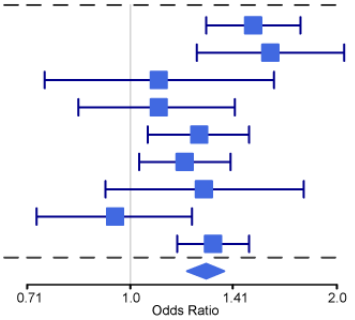

D *HLA* locus

rs9271573	Odds ratio	Lower.limit	Upper.limit	P value
Chinese discovery cohort	3.45	2.9	4.11	3.4E-44
Korean discovery cohort	1.54	1.19	1.98	0.00087
Japanese discovery cohort	1.47	1.03	2.1	0.035
Turkish discovery cohort	1.44	1.13	1.82	0.0029
European discovery cohort 1	2.12	1.8	2.49	3.2E-19
European discovery cohort 2	2.29	2	2.62	4.3E-33
Sardinia discovery cohort	1.66	1.18	2.33	0.0029
GCKD discovery cohort	2.28	1.77	2.94	2.8E-11
Chinese replication cohort	3.76	3.24	4.36	6.3E-68
**All combined**	**2.41**	**2.26**	**2.57**	**2.7E-154**

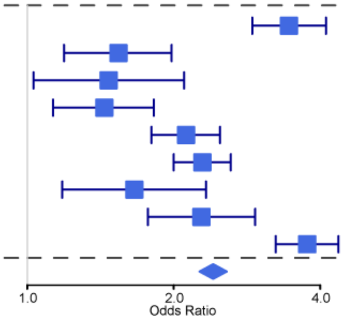

Supplementary Figure 4. Forest plots for the top SNP per each genome-wide significant locus: (A) The association statistics for the top SNP (rs17831251) at the *PLA2R1* locus, including effect estimates (odds ratios), 95% confidence intervals, and p-values for each risk allele; **(B)** *NFKB1* locus (rs230540); **(C)** *IRF4* locus (rs9405192); **(D)** *HLA-DRB1/DQA1* locus (rs9271573). The effect estimates and their corresponding 95% confidence intervals are displayed graphically for each cohort individually and for all cohorts combined.

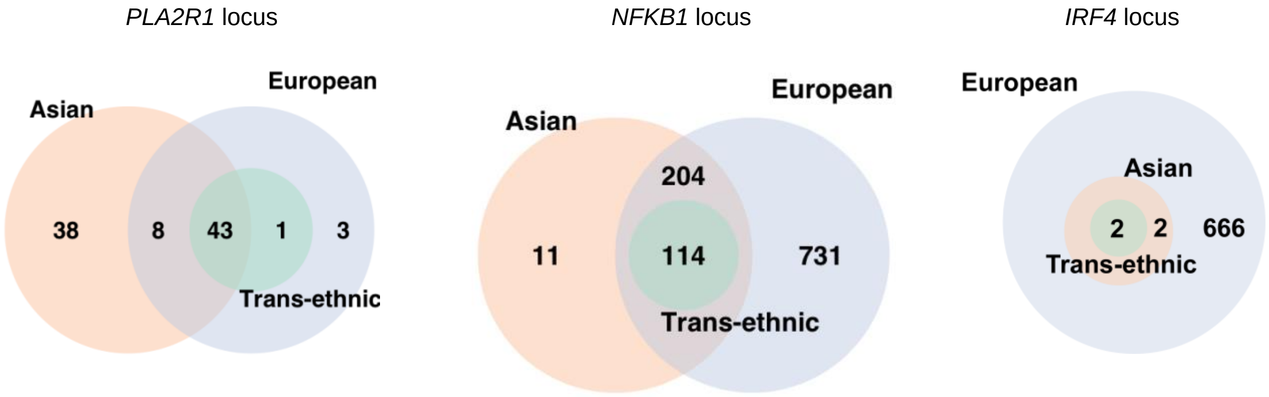

Supplementary Figure 5. Credible sets analysis of the three non-HLA loci. Each circle represents a 99% credible set for the meta-analysis involving East Asian cohorts (orange), European cohorts (blue), and all cohorts combined (green); the exact numbers of SNPs within each set are provided. For each locus, the majority of candidate causal SNPs from transethnic analysis fall at the intersection of ethnicity-specific analyses. In each case, the lead SNP from transethnic meta-analysis is found at the intersection of all three 99% credible sets. In the overall analysis by CAVIAR, the posterior probability for the lead GWAS SNP was 8.4% for the *PLA2R1* locus, 5.1% for the *NFKB1* locus, and 97.5% for the *IRF4* locus. In addition, co-localization analyses of these regions between East Asian and European meta-analyses provided high posterior probability of sharing a single causal variant for each locus (*PLA2R1* locus PP4=94.3%, *NFKB1* locus PP4=78.8%, and *IRF4* locus PP4=64.7% by COLOC analysis).

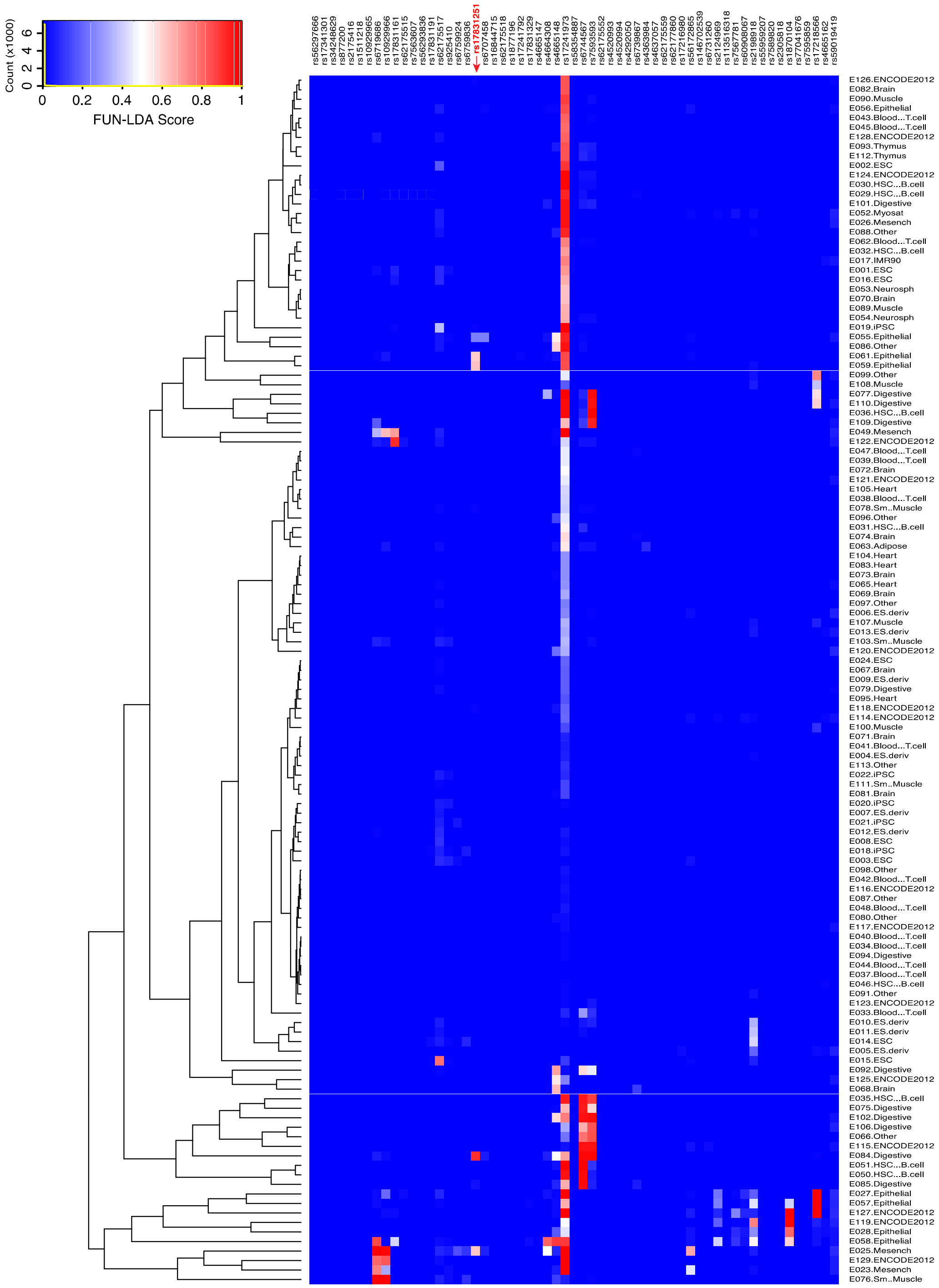

Supplementary Figure 6. Tissue-specific functional predictions for non-coding variants at the *PLA2R1* locus. The index SNP (rs17831251, red arrow) and all its proxies defined by $r_2 > 0.8$ (East Asian and European individuals from 1000G phase 3). The SNPs were ordered by genomic location and the 127 tissues/cell types from Roadmap Epigenomics were clustered using hierarchical clustering approach based on the pattern of functional latent Dirichlet allocation (FUN-LDA) scores. The top-ranking SNP was rs17241973 with functional prediction across multiple tissues.

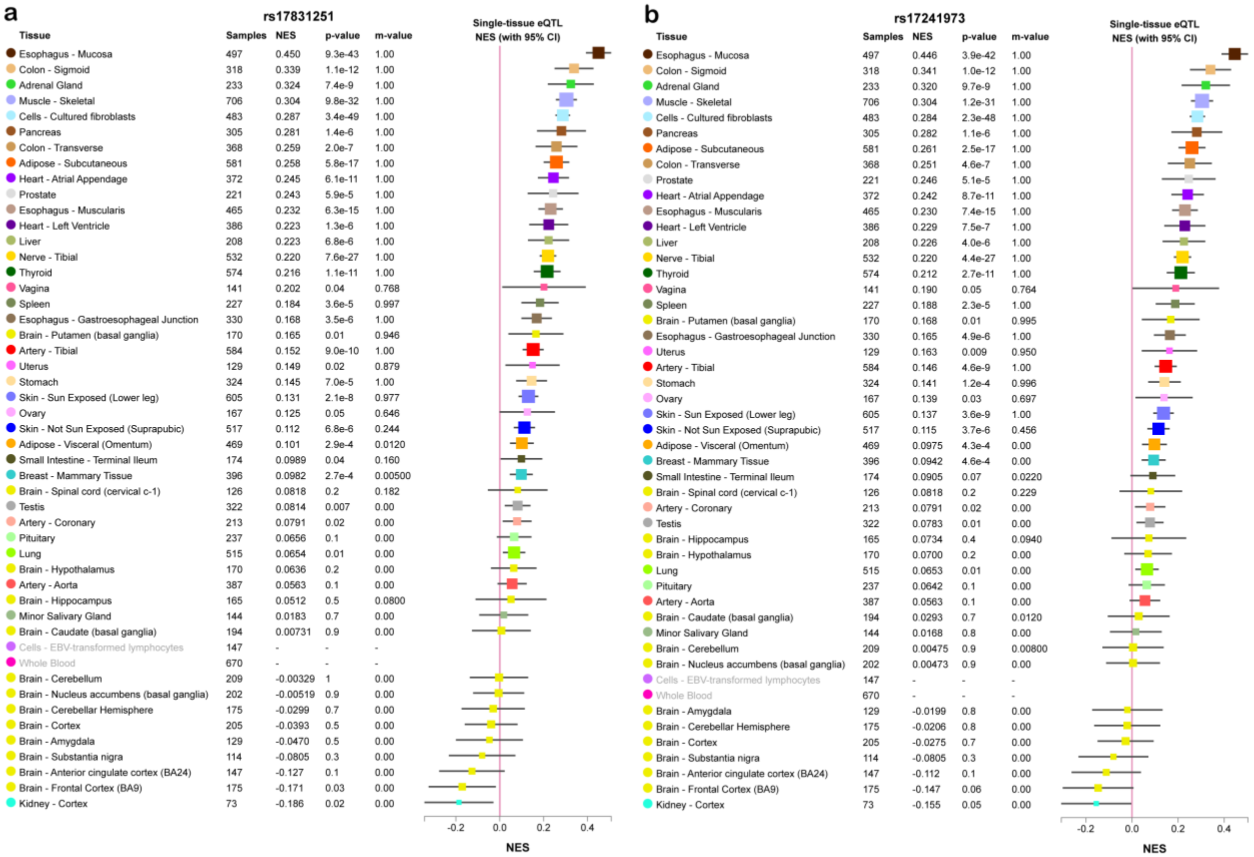

Supplementary Figure 7. Genotype-Tissue Expression (GTEx) multi-tissue analysis of cis-QTL effects on *PLA2R1* gene expression for (a) rs17831251 and (b) rs17241973. The normalized effect size (NES) is defined as the slope of the linear regression, and is computed as the effect of the alternative allele relative to the reference allele in the hg38 human genome reference; eQTL effect allele is the MN protective allele, and the reference allele is the MN risk allele for each SNP; 95% confidence intervals are also provided. The current release of GTEx (V8) contains only 73 kidney cortex samples, but the NES is opposite to all other tissues except for brain, where *PLA2R1* is expressed only weakly.

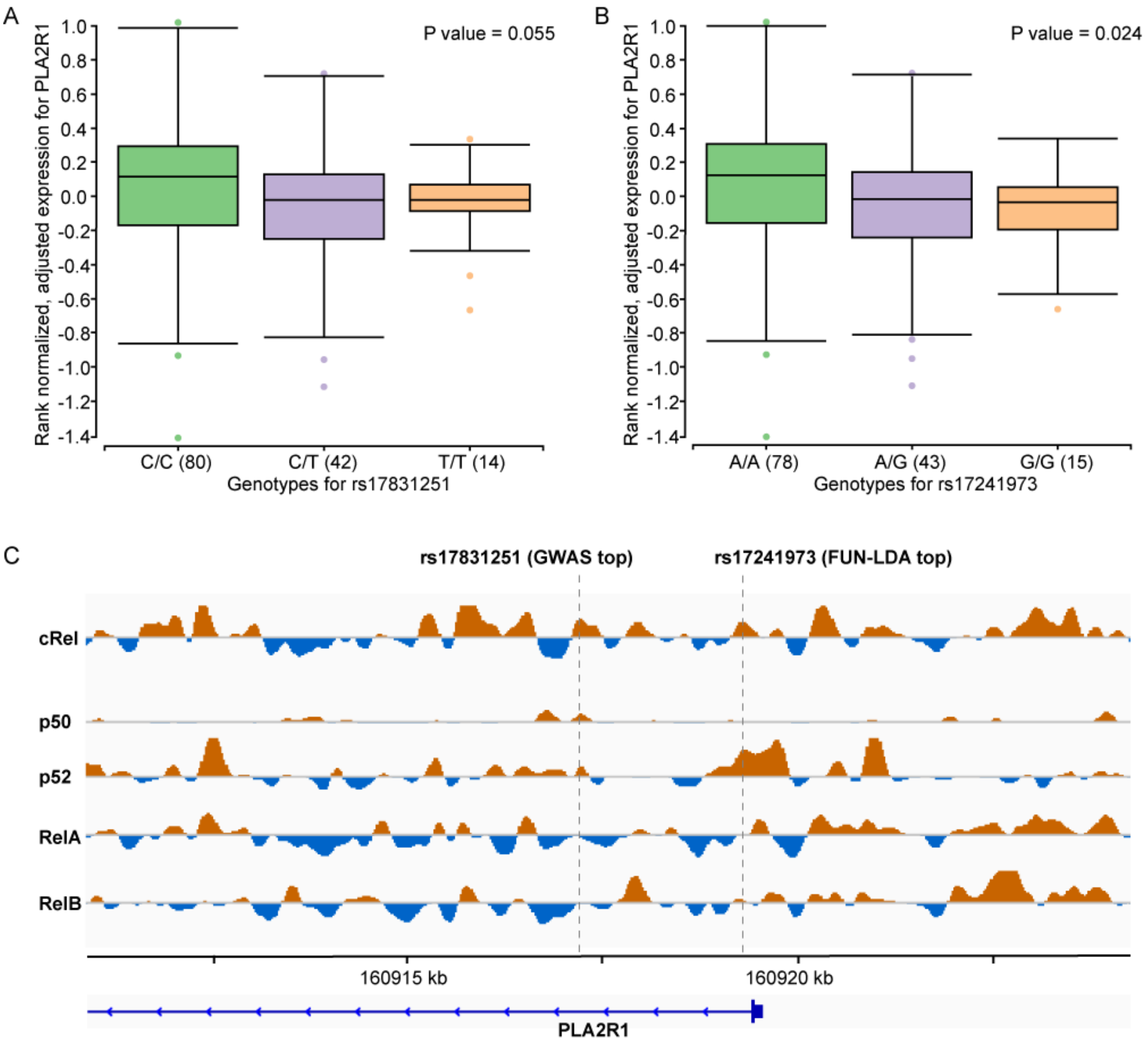

Supplementary Figure 8. Functional annotation of the *PLA2R1* locus. (A) The NEPTUNE cis-eQTL effect of rs17831251 (the index SNP) on *PLA2R1* mRNA expression suggests that the risk allele may be associated with a higher expression of *PLA2R1* in kidney glomeruli (P=0.055); **(B)** The NEPTUNE glomerular cis-eQTL effect of rs17241973 (the top ranking SNP based on FUN-LDA) is stronger and significant (P=0.024) compared to rs17831251; **(C)** Regional plot of Chip-seq peaks for all five subunits of NFκB complex in immortalized lymphocytes; the dash lines correspond to the genomic locations of rs17831251 and rs17241973 in relation to the potential NFκB complex binding sites.

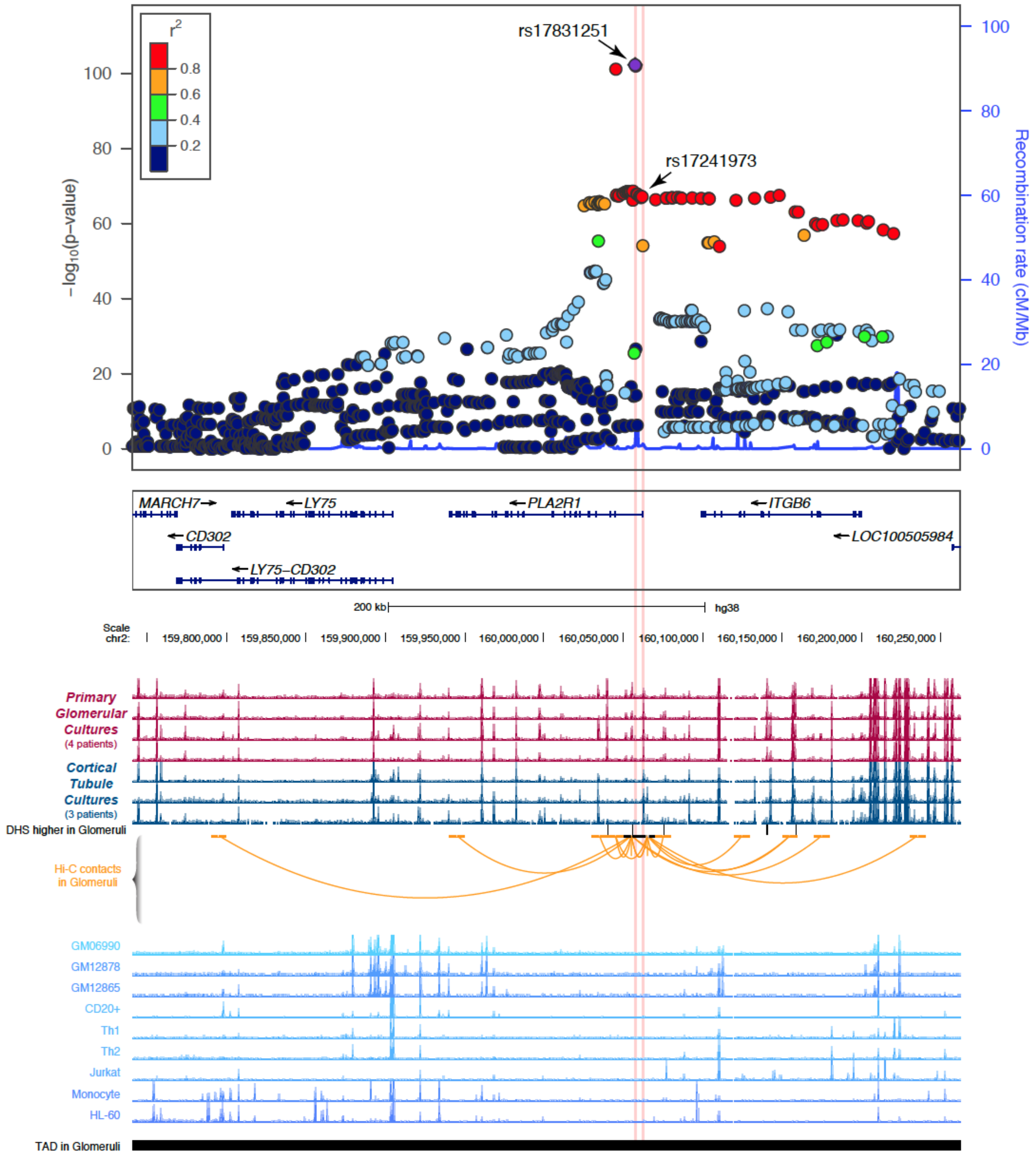

Supplementary Figure 9. Human kidney and immune cell-specific chromatin landscape at the *PLA2R1* locus. The most strongly associated variants span the broader region encoding *PLA2R1* and *ITGB6.* This region overlaps with multiple glomerular compartment-specific Hi-C contact sites forming a composite cis-regulatory module. The nearest DHS to rs17831251 (intron 1), which also has higher accessibility in the glomerular samples, is only 2.1 kb away [chr2:160,055,135-160,055,696 (hg38)] and contains a high confidence *NFKB1* binding motif. The rs17241973 (also in intron 1) intersects a DHS active in both glomeruli and tubules. In contrast, none of these sites appear active in immune cells.

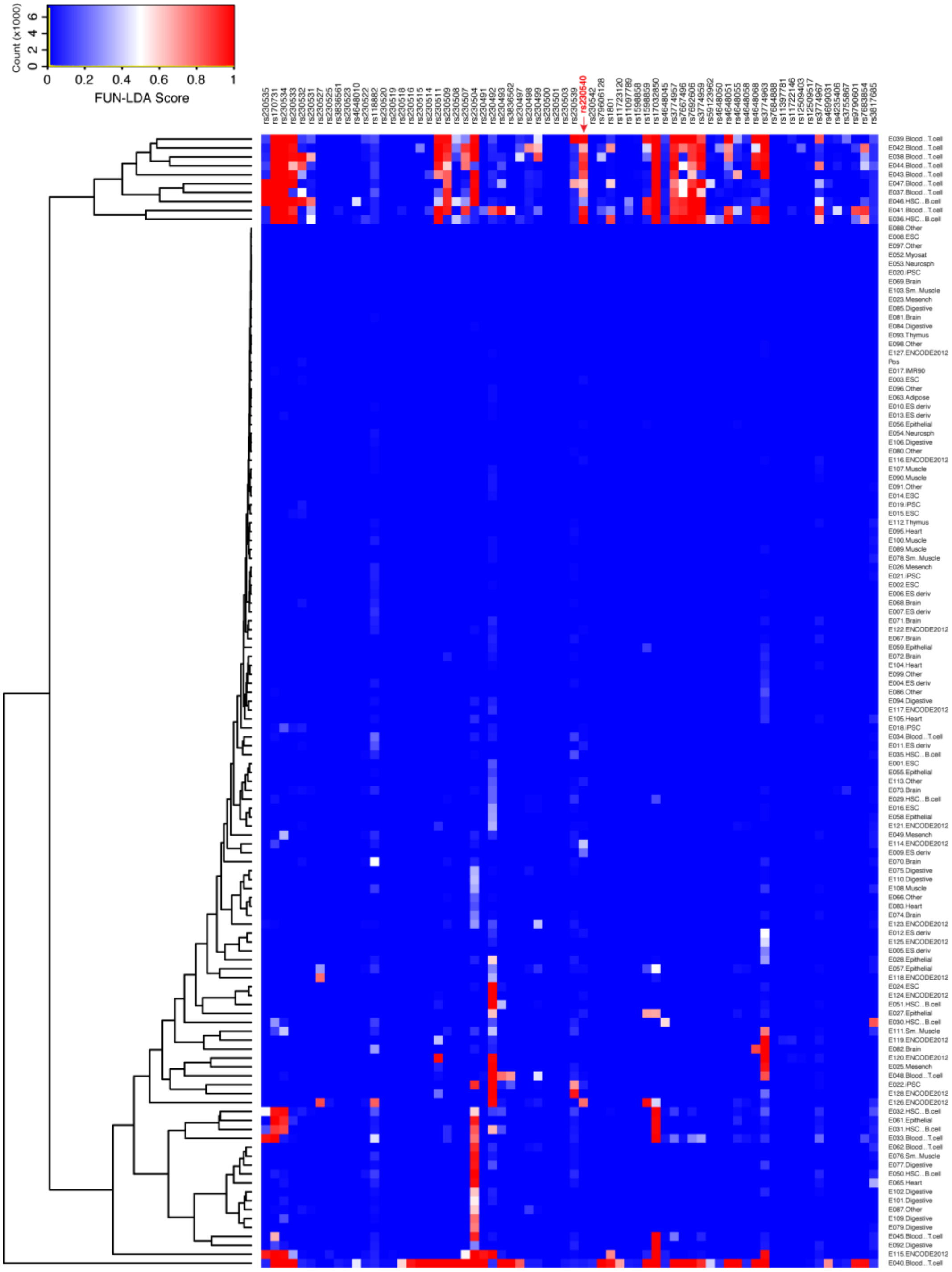

Supplementary Figure 10. Tissue-specific functional predictions for non-coding variants at the *NFKB1* locus. The index SNP (rs230540, red arrow) and its proxies defined by $r^2 > 0.8$ based on East Asian and European individuals from 1000G phase 3. The SNPs were ordered by genomic location and the 127 tissues/cell types from Roadmap Epigenomics were clustered using hierarchical clustering approach based on the pattern of functional latent Dirichlet allocation (FUN-LDA) scores. The majority of predicted functional elements intersected by this signal correspond to primary immune cells (T-cells and B-cells).

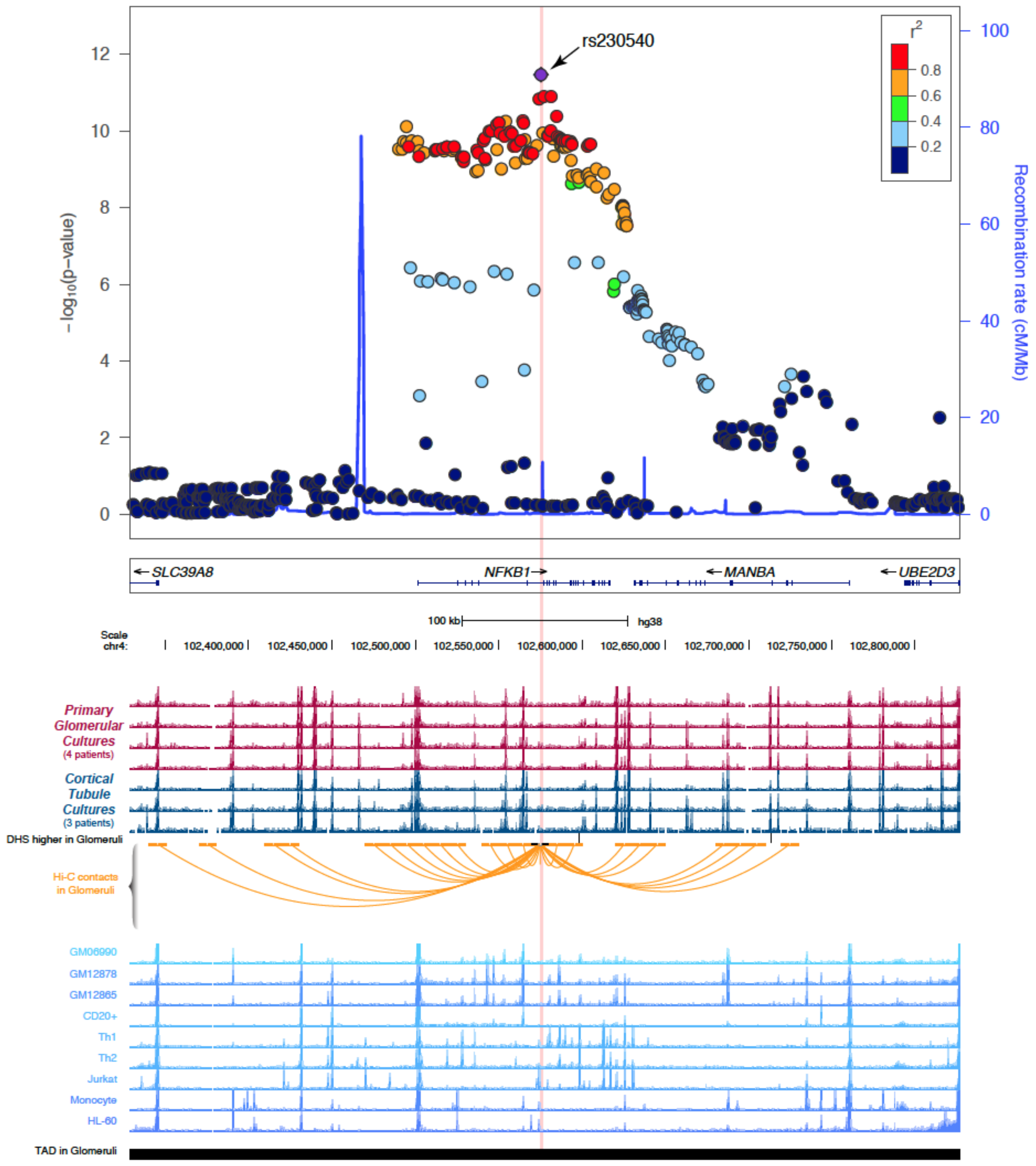

Supplementary Figure 11. Kidney and immune cell-specific chromatin landscape at the *NFKB1* locus. Consistent with FUN-LDA prediction, rs230540 intersects an active DHS in monocytes and T-cells, but also in glomerular cells, and it maps to several glomerular Hi-C contact sites suggesting a functional role of this locus in the kidney.

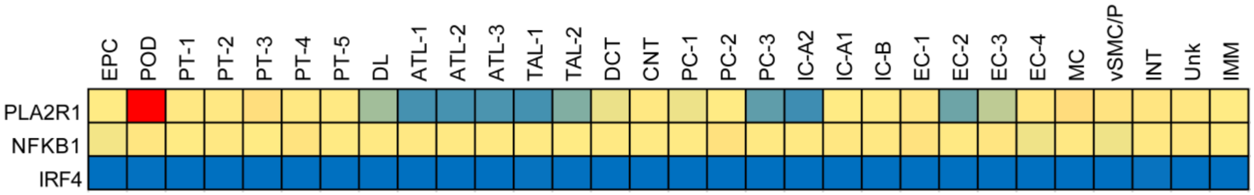

Supplementary Figure 12. Gene expression of *PLA2R1, NFKB1,* and *IRF4* by kidney snRNA-seq[1]. *PLA2R1* is strongly expressed only in podocytes, *NFKB1* is weakly expressed across all different renal cell types, while *IRF4* does not appear to be expressed in any of the renal cell clusters. Color scale: red (strong expression), yellow (weak expression), blue (no expression).

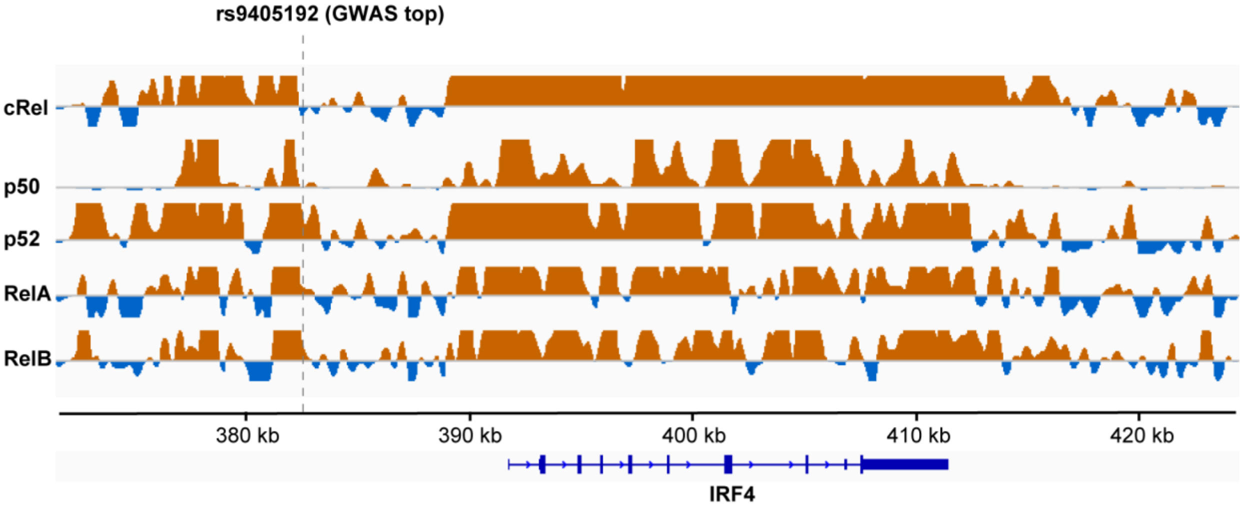

Supplementary Figure 13. Functional annotation of the *IRF4* locus. Reginal plot of Chip-seq peaks for all five subunits of NFκB complex in immortalized lymphocytes; the dash lines correspond to the genomic location of rs9405192, the top SNP at this locus, in relation to the coding sequence of *IRF4* and the NF-κB complex binding sites.

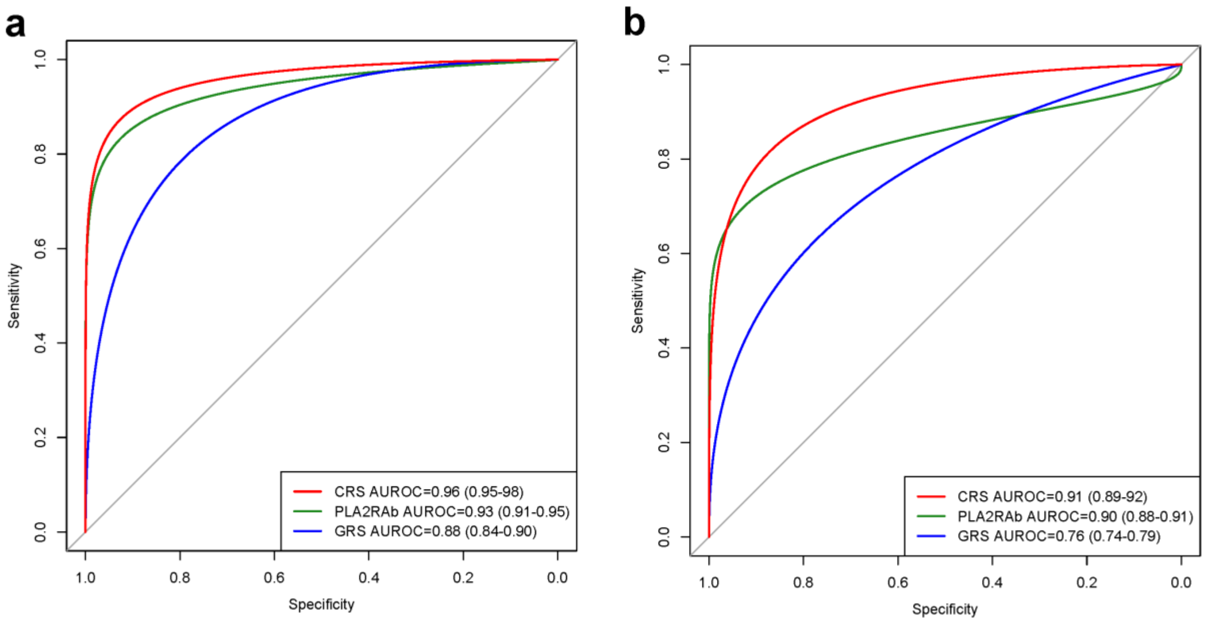

Supplementary Figure 14. Overall comparison of ROC curves for GRS, PLA2R antibody test, and CRS: (a) East Asian cohorts of 304 MN cases vs. 155 controls and **(b)** European cohorts of 1,094 MN cases vs. 695 controls. Only individuals with both serum and genetic data were included in the analysis of GRS and CRS. The standard diagnostic cut-off for the PLA2R Ab test of 20 U/mL provides 100% specificity and 60% sensitivity in East Asians and 100% specificity and 57% sensitivity in Europeans. AUROC: Area under the ROC curve.

a

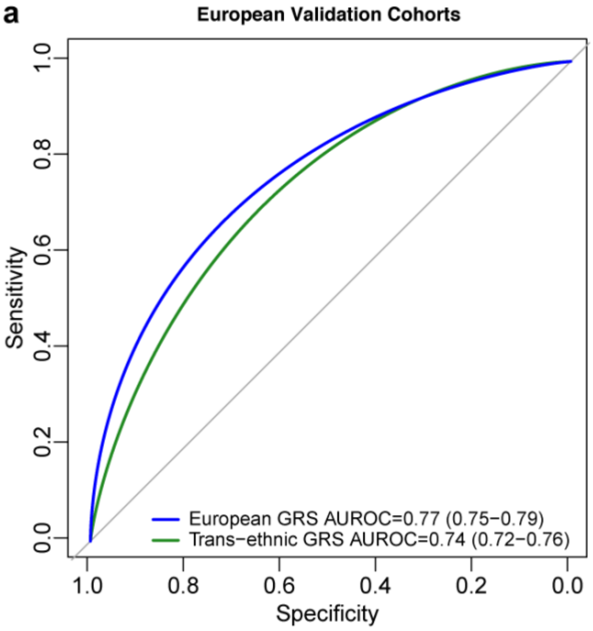

b

European GRS in European Validation Cohorts

European GRS Tail	Reference	Odds Ratio	95% CI	P value
Top 20% of distribution	Remaining 80%	6.65	5.47-8.08	3.7E-81
Top 10% of distribution	Remaining 90%	7.02	5.48-9.00	1.6E-53
Top 5% of distribution	Remaining 95%	9.80	6.09-13.9	1.9E-37
Top 1% of distribution	Remaining 99%	14.8	6.33-34.5	4.6E-10
Top 0.5% of distribution	Remaining 99.5%	16.9	4.75-60.1	1.2E-05

c

Trans-ethnic GRS in European Validation Cohorts

Trans-ethnic GRS Tail	Reference	Odds Ratio	95% CI	P value
Top 20% of distribution	Remaining 80%	3.73	3.73-5.47	1.4E-53
Top 10% of distribution	Remaining 90%	4.58	3.58-5.86	6.4E-34
Top 5% of distribution	Remaining 95%	4.95	3.55-6.89	2.8E-21
Top 1% of distribution	Remaining 99%	5.89	2.87-12.1	1.3E-06
Top 0.5% of distribution	Remaining 99.5%	4.79	1.73-13.3	2.6E-03

Supplementary Figure 15. European GRS has superior predictive performance compared to trans-ethnic GRS in the European validation cohorts, including in the risk stratification by variable upper tail cut-offs of the GRS distribution: (a) Comparison of area under the ROC curve (AUROC) demonstrates improved performance of the European GRS in comparison to the trans-ethnic GRS when applied to the European validation cohorts; **(b)** Odds ratios of disease in upper tails of the European GRS distribution when applied to the European validation cohorts; **(c)** Odds ratios of disease for upper tails of the Trans-ethnic GRS distribution when applied to the European validation cohorts.

Supplementary Table 1. SNPs selected for replication: all SNPs with P-value < 5 x 10^{-5} in the discovery phase were genotyped in the replication cohort; the association results are presented for the discovery phase, replication phase, and joint analysis of all cohorts. Minor allele was used as a test allele to calculate effect sizes. Genome-wide significant SNPs are highlighted in yellow.

RSID	Chr.	Position (Hg19)	Tested allele	Ref allele	Effect	SE	*P* discovery	Effect	SE	*P* replication	Effect	SE	*P* combined	Gene annotation
rs9271573	6	32590501	A	C	0.78	0.04	1.8E-97	0.57	0.08	6.3E-68	0.88	0.03	2.7E-154	*[HLA DRB1-DQA1]*
rs17831251	2	160914156	T	C	-0.72	0.04	1.2E-69	-0.58	0.10	1.2E-42	-0.81	0.04	4.7E-103	*[PLA2R1]*
rs6707458	2	160914540	T	G	-0.72	0.04	1.0E-69	-0.57	0.10	6.3E-42	-0.81	0.04	9.2E-103	*[PLA2R1]*
rs56297666	2	160902023	C	G	-0.71	0.04	1.7E-68	-0.59	0.10	3.8E-43	-0.81	0.04	6.4E-102	*[PLA2R1]*
rs9405192	6	382537	A	G	-0.24	0.04	2.1E-10	-0.12	0.06	1.2E-05	-0.25	0.03	1.4E-14	*[IRF4]*
rs230540	4	103496667	T	C	-0.25	0.04	9.0E-12	-0.06	0.07	4.2E-02	-0.22	0.03	3.4E-12	*[NFKB1]*
rs230542	4	103498455	T	C	0.25	0.04	1.0E-11	0.05	0.07	8.8E-02	0.22	0.03	1.3E-11	*[NFKB1]*
rs6833745	4	103415822	A	C	-0.23	0.04	2.2E-10	-0.06	0.06	4.7E-02	-0.21	0.03	7.6E-11	*[NFKB1]*
rs2125213	4	103411099	A	G	0.23	0.04	2.8E-10	0.05	0.06	9.0E-02	0.20	0.03	2.9E-10	*[NFKB1]*
rs2671427	6	385735	T	C	-0.19	0.04	1.3E-07	-0.09	0.06	8.7E-04	-0.20	0.03	4.5E-10	*[IRF4]*
rs4664288	2	160410755	A	T	0.19	0.04	1.3E-07	0.09	0.06	1.0E-03	0.20	0.03	5.2E-10	*[PLA2R1]*
rs149665	14	72636594	A	C	0.19	0.04	2.1E-07	0.03	0.06	2.8E-01	0.16	0.03	5.3E-07	*[RGS6]*
rs59823810	12	64224210	C	G	0.22	0.04	6.7E-07	0.04	0.07	1.6E-01	0.18	0.04	8.2E-07	*[SRGAP1,TMEM5]*
rs6721570	2	225294852	T	C	0.22	0.05	1.3E-06	0.04	0.08	2.2E-01	0.19	0.04	1.4E-06	*[CUL3,FAM124B]*
rs4912415	1	57255169	C	G	-0.17	0.04	1.7E-05	-0.06	0.08	1.2E-01	-0.16	0.04	5.0E-06	*[C1orf168]*
rs10922324	1	197867405	A	T	-0.22	0.05	3.9E-06	-0.02	0.12	6.3E-01	-0.19	0.04	8.1E-06	*[C1orf53,DENND1B,LHX9]*
rs6860340	5	115852960	A	G	-0.17	0.04	2.7E-05	-0.04	0.07	1.9E-01	-0.15	0.03	2.0E-05	*[SEMA6A]*
rs12909296	15	39371768	T	C	0.40	0.10	3.2E-05	0.10	0.21	2.7E-01	0.37	0.09	2.3E-05	*[]*
rs9348123	6	166771814	T	C	-0.18	0.04	4.1E-05	-0.05	0.12	3.3E-01	-0.17	0.04	2.8E-05	*[MPC1,PRR18,RPS6KA2,SFT2D1]*
rs10252519	7	77091845	C	G	-0.15	0.04	3.4E-05	-0.03	0.07	2.9E-01	-0.14	0.03	3.1E-05	*[GCNT1P5]*
rs71431137	2	111817805	T	C	-0.27	0.06	7.2E-06	0.02	0.16	7.4E-01	-0.23	0.06	4.3E-05	*[ACOXL,LOC400997]*
rs12138326	1	14845459	A	G	0.17	0.04	2.9E-05	0.02	0.09	5.8E-01	0.14	0.04	5.5E-05	*[]*
rs4802709	19	50995880	A	G	0.21	0.04	2.6E-07	-0.08	0.09	5.1E-02	0.14	0.04	9.0E-05	*[ASPDH,EMC10,FAM71E1,JOSD2,LRRC4B]*
rs11088004	21	27931795	T	C	0.36	0.08	2.5E-06	-0.01	0.13	8.1E-01	0.26	0.07	9.1E-05	*[CYYR1]*
rs76054445	2	58835247	A	G	-0.55	0.13	1.2E-05	-0.02	0.21	8.1E-01	-0.42	0.11	1.0E-04	*[LINC01122]*
rs3018404	11	132602735	T	G	0.15	0.04	3.4E-05	0.01	0.07	6.5E-01	0.12	0.03	1.1E-04	*[OPCML]*
rs72745632	15	80719445	C	G	-0.17	0.04	2.7E-05	-0.01	0.07	7.1E-01	-0.13	0.03	1.4E-04	*[ARNT2]*
rs4140827	12	16675898	A	C	-0.17	0.04	3.2E-05	0.01	0.09	9.0E-01	-0.14	0.04	1.6E-04	*[LMO3]*
rs6750918	2	11253301	T	C	0.15	0.03	2.7E-05	0.00	0.06	9.1E-01	0.11	0.03	1.9E-04	*[C2orf50,FLJ33534]*
rs485501	8	93511068	C	G	-0.23	0.05	1.2E-05	-0.01	0.07	8.1E-01	-0.16	0.04	1.9E-04	*[]*
rs552879	11	117348907	T	C	-0.17	0.04	3.2E-06	0.02	0.06	4.5E-01	-0.11	0.03	2.1E-04	*[DSCAML1]*
rs9298595	8	40306342	T	C	0.16	0.04	2.5E-05	-0.01	0.08	8.5E-01	0.12	0.03	2.3E-04	*[ZMAT4]*
rs1042357	17	6905061	T	G	-0.15	0.03	2.4E-05	0.00	0.06	8.6E-01	-0.11	0.03	3.0E-04	*ALOX12-AS1*
rs17364498	8	5698503	A	G	0.16	0.04	4.6E-05	-0.01	0.09	7.2E-01	0.13	0.03	3.3E-04	*[]*
rs4945839	6	110132871	A	G	-0.17	0.04	1.6E-05	0.01	0.07	6.8E-01	-0.12	0.03	3.7E-04	*[FIG4]*
rs111752367	2	181363532	T	C	-0.16	0.04	1.8E-05	0.01	0.06	7.6E-01	-0.11	0.03	3.8E-04	*[]*
rs1692144	1	147281349	T	C	-0.21	0.05	3.8E-05	0.04	0.12	4.2E-01	-0.16	0.05	4.7E-04	*[GJA5]*
rs139458785	7	1423966	A	G	0.37	0.09	2.2E-05	-0.07	0.18	3.6E-01	0.27	0.08	5.7E-04	*[INTS1,MAFK,MICALL2,PSMG3,TMEM184A]*
rs35801335	1	161716885	A	G	0.31	0.07	2.1E-05	-0.08	0.16	2.8E-01	0.23	0.07	5.9E-04	*[ATF6,DUSP12,FCRLB]*
rs780375	3	59737251	C	G	0.18	0.04	4.7E-05	-0.07	0.12	1.5E-01	0.13	0.04	9.5E-04	*[FHIT]*
rs61803846	1	164638458	T	C	0.36	0.09	3.5E-05	0.00	0.11	9.5E-01	0.22	0.07	1.2E-03	*[PBX1]*
rs10195618	2	21482292	A	G	0.27	0.06	1.2E-05	-0.09	0.12	1.1E-01	0.18	0.06	1.4E-03	*[]*
rs1016552	3	65379467	T	C	-0.24	0.06	1.1E-05	0.02	0.07	5.6E-01	-0.13	0.04	2.3E-03	*[MAGI1]*
rs74233415	10	67410034	C	G	0.29	0.07	1.0E-05	-0.05	0.10	2.4E-01	0.17	0.05	2.6E-03	*[LINC01515]*
rs1437624	5	122376331	T	C	-0.18	0.04	4.1E-05	0.05	0.06	7.7E-02	-0.09	0.04	1.5E-02	*[PPIC,SNX2,SNX24]*
rs12055325	5	122368738	A	T	-0.18	0.04	4.7E-05	0.05	0.06	5.6E-02	-0.08	0.04	2.0E-02	*[PPIC]*

Supplementary Table 2. Stepwise conditional analyses defined independent SNPs within the HLA region. The conditional analyses were performed using stratified meta-analysis approach separately for East Asian, European, and all cohorts combined.

			Unconditioned			Mutually conditioned		
**Cohorts**	**SNP**	**Risk allele**	**Effect**	**StdErr**	**P value**	**Effect**	**StdErr**	**P value**
**All cohorts**	rs28383314 (HLA1)	T	0.86	0.04	3.08E-117	0.82	0.04	9.36E-93
	rs9273325 (HLA2)	A	0.92	0.05	2.96E-90	0.69	0.05	2.74E-43
	rs3135335 (HLA3)	G	0.51	0.04	1.26E-34	0.33	0.04	1.57E-19
**East Asian**	rs9269027 (HLA1)	A	1.35	0.08	2.32E-67	1.54	0.07	3.98E-97
	rs1974461 (HLA2)	T	0.53	0.21	9.43E-03	1.34	0.21	1.71E-10
**European**	rs9271541 (HLA1)	C	1.01	0.05	2.19E-104	0.88	0.05	5.68E-62
	rs9265949 (HLA2)	T	1.29	0.06	1.08E-90	0.66	0.08	7.73E-18
	rs2858309 (HLA3)	C	0.58	0.04	1.57E-40	0.31	0.05	1.77E-09

Supplementary Table 3. Results of stepwise conditional analyses of classical 4-digit HLA-DRB1 and HLA-DQA1 alleles in East Asians. Independent alleles highlighted in yellow.

Classical HLA-DQA1 and HLA-DRB1 allleles (4-digit resolution)	Freq. Cases	Freq. Controls	Unconditioned			Conditioned on DRB1*1501			Conditioned on DRB1*1501 and DRB1*0301		
			OR	StdErr	P value	OR	StdErr	P value	OR	StdErr	P value
*HLA-DRB1*01:01*	0.01	0.04	0.52	0.22	3.3E-03	0.60	0.23	2.4E-02	0.61	0.23	3.0E-02
*HLA-DRB1*03:01*	0.12	0.03	3.50	0.13	9.2E-23	**3.88**	**0.13**	**4.5E-24**			
*HLA-DRB1*04:05*	0.04	0.08	0.56	0.14	6.2E-05	0.69	0.15	1.3E-02	0.70	0.15	2.0E-02
*HLA-DRB1*07:01*	0.04	0.07	0.53	0.14	5.5E-06	0.63	0.14	1.5E-03	0.72	0.15	2.4E-02
*HLA-DRB1*08:02*	0.02	0.02	1.02	0.23	9.4E-01	1.20	0.24	4.4E-01	1.22	0.24	4.1E-01
*HLA-DRB1*08:03*	0.05	0.07	0.70	0.14	9.2E-03	0.89	0.14	4.1E-01	0.99	0.14	9.2E-01
*HLA-DRB1*09:01*	0.07	0.14	0.38	0.11	6.6E-18	0.50	0.12	1.6E-09	0.54	0.12	1.7E-07
*HLA-DRB1*10:01*	0.01	0.01	0.48	0.37	4.9E-02	0.58	0.38	1.4E-01	0.57	0.39	1.6E-01
*HLA-DRB1*12:01*	0.03	0.05	0.72	0.16	4.4E-02	0.89	0.17	4.8E-01	0.92	0.17	6.2E-01
*HLA-DRB1*12:02*	0.06	0.05	0.94	0.13	6.6E-01	1.21	0.14	1.7E-01	1.42	0.14	1.3E-02
*HLA-DRB1*13:01*	0.01	0.01	0.87	0.29	6.2E-01	0.93	0.30	7.9E-01	1.00	0.30	1.0E+00
*HLA-DRB1*13:02*	0.03	0.06	0.63	0.17	5.3E-03	0.74	0.17	7.2E-02	0.78	0.17	1.6E-01
*HLA-DRB1*15:01*	0.26	0.08	**3.81**	**0.09**	**2.0E-49**						
*HLA-DRB1*15:02*	0.03	0.05	0.81	0.17	2.3E-01	0.99	0.18	9.8E-01	1.03	0.18	8.7E-01
*HLA-DRB1*16:02*	0.01	0.01	0.72	0.27	2.1E-01	0.88	0.27	6.3E-01	1.01	0.28	9.7E-01
*HLA-DQA1*01:01*	0.10	0.11	1.01	0.10	9.5E-01	1.28	0.10	1.4E-02	1.46	0.10	2.4E-04
*HLA-DQA1*01:02*	0.30	0.17	2.14	0.07	1.3E-24	0.75	0.13	2.7E-02	0.81	0.13	1.1E-01
*HLA-DQA1*01:03*	0.08	0.12	0.77	0.11	1.7E-02	0.97	0.11	7.7E-01	1.08	0.11	5.1E-01
*HLA-DQA1*02:01*	0.04	0.06	0.49	0.15	1.3E-06	0.60	0.15	9.7E-04	0.71	0.15	2.4E-02
*HLA-DQA1*03:01*	0.17	0.34	0.43	0.08	1.4E-27	0.55	0.08	1.1E-13	**0.61**	**0.08**	**1.3E-09**
*HLA-DQA1*04:01*	0.01	0.02	0.83	0.28	5.1E-01	1.03	0.28	9.2E-01	1.08	0.29	7.8E-01
*HLA-DQA1*05:01*	0.24	0.14	1.83	0.08	6.8E-14	2.21	0.09	2.2E-20	1.60	0.10	3.8E-06
*HLA-DQA1*06:01*	0.06	0.05	0.98	0.13	8.7E-01	1.25	0.14	1.1E-01	1.51	0.14	3.3E-03

Supplementary Table 4. Stepwise conditional analysis of single amino acid sites within *HLA-DRB1* and *-DQA1* in East Asians: multi-degree of freedom (df) test statistics χ_2 and p-values.

	No Conditioning			Conditioned on Position 13			Conditioned on Positions 13 and 71		
Position	df	χ^2	p value	df	χ^2	p value	Df	χ^2	p value
**HLA-DRB1 amino acids**									
-25	1	17.48735479	2.89E-05	2	0.270614666	0.87	2	0.239465599	0.89
-24	1	46.62043104	8.62E-12						
-17	1	111.7156907	4.12E-26	1	0.143453574	0.70	1	0.053817823	0.82
-16	1	17.46896228	2.92E-05	2	0.270614666	0.87	2	0.239465599	0.89
-1	1	175.2472068	5.29E-40	1	19.42874322	1.04E-05			
4	1	124.1258816	7.91E-29	2	0.415528684	0.81	2	0.248588659	0.88
9	2	112.7421699	3.30E-25	2	0.291400688	0.86	2	0.151800352	0.93
10	2	25.26455682	3.26E-06	1	0.005193518	0.94	1	0.001843865	0.97
11	5	282.6934332	5.25E-59	2	0.291400688	0.86	2	0.151800352	0.93
12	1	21.4193576	3.69E-06						
13	5	317.9339469	1.39E-66						
14	1	24.94426148	5.90E-07						
16	1	7.056649476	7.90E-03						
25	1	24.99413789	5.75E-07						
26	2	19.71271422	5.24E-05	3	34.62503693	1.46E-07	2	0.850772617	0.65
28	2	155.0906748	2.10E-34	2	0.255828134	0.88	2	0.134679459	0.93
30	5	165.1108164	8.05E-34	3	0.292172008	0.96	3	0.155675091	0.98
31	2	111.555223	5.97E-25	1	0.005193518	0.94	1	0.001843865	0.97
32	1	20.86850611	4.92E-06	3	3.790037248	0.29	3	6.54909177	0.09
33	1	46.82798705	7.75E-12						
37	4	99.81053135	1.08E-20	10	8.055012077	0.62	9	8.595251233	0.48
38	2	8.850889733	1.20E-02	2	0.005195066	1.00	2	0.009813358	1.00
40	1	6.033304342	1.40E-02	1	0.005193518	0.94	1	0.001843865	0.97
47	1	197.0051765	9.41E-45	3	0.267772925	0.97	2	1.078616241	0.58
57	2	178.3311662	1.89E-39	8	2.141528559	0.98	8	1.116836568	1.00
60	1	95.90620488	1.20E-22	5	0.07562306	1.00	5	0.109945774	1.00
67	2	36.99965021	9.24E-09	6	29.01982627	6.03E-05	5	11.83054238	0.04
70	2	94.18371999	3.53E-21	6	77.89520241	9.72E-15	5	9.435752427	0.09
71	3	265.9582767	2.31E-57	4	87.67483758	4.10E-18			
73	1	13.04286327	3.04E-04	1	45.35872458	1.64E-11			
74	4	164.7075531	1.43E-34	7	53.77593227	2.60E-09	6	7.562704698	0.27
77	1	98.5821584	3.12E-23	1	45.35872458	1.64E-11			
78	1	123.8124506	9.26E-29	1	0.255080136	0.61	1	0.130762042	0.72
85	1	3.241012195	7.18E-02	1	1.44897E-06	1.00	1	0.007973799	0.93
86	1	211.6839029	5.90E-48	5	72.53585558	3.04E-14	7	32.87325248	2.80E-05
96	3	167.9127537	3.59E-36	6	1.032947621	0.98	6	0.675006328	1.00
98	1	193.6913863	4.97E-44	2	0.244701277	0.88	2	0.131413267	0.94
104	1	194.1028071	4.04E-44	2	0.244701277	0.88	2	0.131413267	0.94
120	1	52.75865819	3.77E-13	2	0.029060816	0.99	2	0.020780823	0.99
133	1	160.8720313	7.30E-37	2	3.643650591	0.16	2	2.828603422	0.24
140	1	0.314188762	5.75E-01	3	0.259114802	0.97	3	0.174660737	0.98
142	1	160.8826555	7.26E-37	2	3.643650591	0.16	2	2.828603422	0.24
149	1	20.08871274	7.39E-06	3	2.285300613	0.52	3	1.627854715	0.65
166	1	5.290240803	2.14E-02	1	0.005193518	0.94	1	0.001843865	0.97
180	1	46.15995557	1.09E-11	2	0.046730376	0.98	2	0.046497382	0.98
181	1	130.9836533	2.50E-30	1	0.33783095	0.56	1	0.204106393	0.65
189	1	6.072342204	1.37E-02	1	1.74783333	0.19	1	2.025600169	0.15
231	1	6.256358585	1.24E-02	1	0.085102405	0.77	1	0.071106369	0.79
233	1	37.31047205	1.01E-09	3	1.066736419	0.79	3	1.393964272	0.71
**HLA-DQA1 amino acids**									
-16	1	48.92488943	2.66E-12	5	6.679625594	2.46E-01	5	3.446636572	6.31E-01
11	1	54.61131395	1.47E-13	5	9.952237269	7.66E-02	5	0.769766102	9.79E-01
18	1	54.61131395	1.47E-13	5	9.952237269	7.66E-02	5	0.769766102	9.79E-01
25	1	22.95926698	1.65E-06	4	16.30149866	2.64E-03	5	12.45989381	2.90E-02
26	1	124.6860277	5.96E-29	3	0.170547032	9.82E-01	3	0.140353078	9.87E-01
34	1	150.7680924	1.18E-34	4	2.54339509	6.37E-01	4	1.81395041	7.70E-01
40	1	36.92774373	1.23E-09	3	11.77489915	8.20E-03	3	0.912278889	8.22E-01
41	1	5.796725317	1.61E-02	3	23.80486841	2.74E-05	4	20.41538525	4.13E-04
45	1	54.61131395	1.47E-13	5	9.952237269	7.66E-02	5	0.769766102	9.79E-01
47	3	168.6229346	2.52E-36	7	14.25219946	4.69E-02	7	2.391263603	9.35E-01
48	1	54.61131722	1.47E-13	5	9.952236699	7.66E-02	5	0.769766102	9.79E-01
50	2	168.6382593	2.40E-37	7	11.97824979	1.01E-01	7	0.949943601	9.96E-01
51	1	36.92774373	1.23E-09	3	11.77489915	8.20E-03	3	0.912278889	8.22E-01
52	2	65.88573939	4.93E-15	5	13.38306546	2.00E-02	5	1.876659881	8.66E-01
53	2	168.6382593	2.40E-37	7	11.97824979	1.01E-01	7	0.949943601	9.96E-01
54	1	23.77978142	1.08E-06	1	1.838773819	1.75E-01	1	1.382881693	2.40E-01
55	1	54.61131395	1.47E-13	5	9.952237269	7.66E-02	5	0.769766102	9.79E-01
56	2	127.2212724	2.37E-28	7	10.63660091	1.55E-01	7	1.447466904	9.84E-01
61	1	54.61131722	1.47E-13	5	9.952236699	7.66E-02	5	0.769766102	9.79E-01
64	1	54.61131395	1.47E-13	5	9.952237269	7.66E-02	5	0.769766102	9.79E-01
66	1	54.61131722	1.47E-13	5	9.952236699	7.66E-02	5	0.769766102	9.79E-01
69	2	55.86527002	7.40E-13	6	9.962015795	1.26E-01	6	4.003534195	6.76E-01
75	1	52.10398322	5.26E-13	3	9.015334121	2.91E-02	3	4.730026085	1.93E-01
76	2	127.2212724	2.37E-28	7	10.63660091	1.55E-01	7	1.447466904	9.84E-01
80	1	54.61131722	1.47E-13	5	9.952236699	7.66E-02	5	0.769766102	9.79E-01
107	1	52.10398322	5.26E-13	3	9.015334121	2.91E-02	3	4.730026085	1.93E-01
129	1	87.01875081	1.07E-20	5	0.489949272	9.92E-01	6	20.53820325	2.22E-03
130	1	5.808751006	1.59E-02	3	23.8525038	2.68E-05	4	20.4590194	4.05E-04
156	1	52.10398322	5.26E-13	3	9.015334121	2.91E-02	3	4.730026085	1.93E-01
161	1	52.10398322	5.26E-13	3	9.015334121	2.91E-02	3	4.730026085	1.93E-01
163	1	52.10398322	5.26E-13	3	9.015334121	2.91E-02	3	4.730026085	1.93E-01
175	2	170.1278204	1.14E-37	7	11.2342691	1.29E-01	7	4.921576696	6.70E-01
187	1	124.7706228	5.71E-29	3	0.171502632	9.82E-01	3	0.141120239	9.86E-01
207	1	106.7830764	4.97E-25	4	0.388197468	9.83E-01	4	26.88674976	2.10E-05
215	1	168.0813471	1.94E-38	4	0.001917111	1.00E+00	4	0.000645567	1.00E+00
218	1	54.65073982	1.44E-13	5	9.966067868	7.62E-02	5	0.771191363	9.79E-01

Supplementary Table 5. Effects of individual amino acid residues at positions 13 and 71 of the *HLA-DRB1* gene in East Asians.

Amino acid residuals		Freq. Cases	Freq. Controls	Odds Ratio	95%CI
HLA-DRB1 position 13	Arg	0.30	0.14	3.68	2.74-4.95
	Ser	0.31	0.20	2.76	2.06-3.71
	Gly	0.18	0.22	1.57	1.16-2.13
	His	0.09	0.18	1.05	0.75-1.45
	Tyr	0.04	0.07	1.00	*Reference*
	Phe	0.09	0.20	0.79	0.57-1.09
HLA-DRB1 position 71	Lys	0.13	0.05	3.10	2.49-3.86
	Ala	0.29	0.13	2.96	2.55-3.45
	Arg	0.55	0.75	1.00	*Reference*
	Glu	0.04	0.07	0.91	0.68-1.22

Supplementary Table 6. Haplotypes defined by *HLA-DRB1* positions 13 and 71 in East Asians. A total of 10 East Asian haplotypes were defined by these two amino acid positions. We chose one haplotype which has similar frequencies in cases and controls as references to compute odds ratios for all other haplotypes. We list unadjusted haplotype frequencies for cases and controls, and the four-digit HLA-DRB1 classical alleles tagged by each haplotype.

HLA-DRB1 amino acid at position				Allele frequency		
13	71	OR	95% CI	Controls	Cases	Classical HLA-DRB1 alleles
**Ser**	**Lys**	**3.57**	**2.73-4.67**	**0.031**	**0.119**	***03:01**
**Arg**	**Ala**	**3.31**	**2.70-4.04**	**0.084**	**0.257**	***15:01**
Ser	Arg	1.79	1.44-2.21	0.093	0.148	*Other*
Gly	Arg	1.00	*Reference*	0.219	0.182	*Other*
Arg	Ala	0.90	0.63-1.29	0.046	0.029	*Other*
Arg	Arg	0.84	0.49-1.44	0.015	0.012	*Other*
Ser	Glu	0.77	0.56-1.05	0.071	0.039	*Other*
His	Lys	0.70	0.37-1.30	0.014	0.008	*Other*
Tyr	Arg	0.67	0.49-0.90	0.071	0.043	*Other*
His	Arg	0.65	0.51-0.82	0.161	0.078	*Other*
Phe	Arg	0.53	0.35-0.80	0.052	0.019	*Other*
Phe	Arg	0.50	0.39-0.64	0.143	0.067	**09:01*

Supplementary Table 7. Results of stepwise conditional analyses of classical 4-digit alleles of HLA-DRB1 and HLA-DQA1 in Europeans.

Classical Alleles (4-digit resolution)	Freq. Cases	Freq. Controls	Unconditioned			Conditioned on DQA1*05:01			Conditioned on DQA1*05:01 and DRB1*03:01		
			OR	SE	P value	OR	SE	P value	OR	SE	P value
*HLA-DQA1*05:01*	0.53	0.30	**2.88**	**0.05**	**5.7E-93**						
*HLA-DRB1*03:01*	0.21	0.12	3.39	0.06	5.2E-82	**2.00**	**0.08**	**2.0E-19**			
*HLA-DRB1*07:01*	0.08	0.13	0.50	0.08	1.8E-18	0.72	0.08	6.0E-05	0.71	0.08	5.0E-05
*HLA-DQA1*02:01*	0.08	0.13	0.50	0.08	2.3E-18	0.72	0.08	7.3E-05	0.72	0.08	6.2E-05
*HLA-DQA1*01:02*	0.11	0.18	0.62	0.06	1.3E-13	0.94	0.07	3.7E-01	0.95	0.07	5.0E-01
*HLA-DQA1*04:01*	0.01	0.03	0.33	0.18	2.0E-09	0.49	0.19	1.4E-04	0.49	0.19	1.8E-04
*HLA-DRB1*01:01*	0.04	0.08	0.57	0.09	2.3E-09	0.84	0.10	6.8E-02	0.83	0.10	5.6E-02
*HLA-DRB1*15:01*	0.05	0.10	0.69	0.08	3.7E-06	1.02	0.08	8.0E-01	1.03	0.08	7.0E-01
*HLA-DRB1*13:02*	0.03	0.04	0.56	0.13	1.5E-05	0.81	0.14	1.3E-01	0.86	0.14	2.9E-01
*HLA-DQA1*03:01*	0.10	0.15	0.78	0.06	5.6E-05	1.22	0.07	3.4E-03	1.23	0.07	2.9E-03
*HLA-DQA1*01:03*	0.04	0.07	0.69	0.09	7.5E-05	1.05	0.10	6.5E-01	1.04	0.10	6.9E-01
*HLA-DRB1*13:01*	0.04	0.05	0.69	0.10	1.7E-04	1.03	0.10	7.6E-01	1.03	0.11	7.8E-01
*HLA-DQA1*01:01*	0.11	0.14	0.78	0.07	2.8E-04	1.23	0.07	5.0E-03	1.21	0.07	1.1E-02
*HLA-DRB1*13:03*	0.01	0.02	0.68	0.21	6.8E-02	0.35	0.22	2.1E-06	0.49	0.22	1.5E-03
*HLA-DRB1*12:01*	0.02	0.01	1.28	0.17	1.5E-01	0.68	0.18	2.9E-02	0.91	0.18	6.2E-01

Supplementary Table 8. Stepwise conditional analysis of single amino acid sites within *DRB1* and *DQA1* in Europeans: multi-degree of freedom (df) test statistics χ_2 and p-values.

	No conditioning			Conditioned on *DQA1* position 75			Conditioned on *DQA1* pos. 75 and *DRB1* pos. 74		
Position	df	χ^2	p value	df	χ^2	p value	Df	χ^2	p value
HLA-DRB1 amino acids									
-25	2	305.09	5.62E-67	4	36.08	2.79E-07	7	7.55	3.74E-01
-24	2	41.34	1.06E-09	4	30.41	4.03E-06	7	7.91	3.41E-01
-17	2	112.32	4.06E-25	4	29.88	5.17E-06	6	11.23	8.16E-02
-16	2	305.09	5.62E-67	4	36.08	2.79E-07	7	7.55	3.74E-01
-1	2	48.70	2.66E-11	4	26.90	2.09E-05	6	6.72	3.47E-01
4	1	68.62	1.19E-16	1	8.29	3.98E-03	1	0.60	4.39E-01
9	2	42.78	5.14E-10	2	1.21	5.46E-01			
9	2	191.15	3.10E-42	3	19.57	2.08E-04	3	4.96	1.75E-01
10	2	251.21	2.82E-55	3	3.12	3.73E-01	4	1.01	9.09E-01
11	5	270.70	1.98E-56	8	26.12	1.00E-03	8	5.38	7.16E-01
12	1	250.44	2.08E-56	2	2.25	3.25E-01	3	0.45	9.30E-01
13	5	310.63	5.20E-65	9	32.33	1.75E-04	11	2.63	9.95E-01
14	1	78.12	9.70E-19	1	14.76	1.22E-04			
25	1	78.12	9.70E-19	1	14.76	1.22E-04			
26	2	355.58	6.11E-78	4	74.53	2.50E-15	5	2.83	7.26E-01
28	2	105.23	1.41E-23	3	22.70	4.67E-05	4	2.40	6.62E-01
30	5	125.59	2.05E-25	7	28.00	2.20E-04	6	3.57	7.34E-01
31	2	27.56	1.04E-06	3	0.71	8.70E-01	4	1.84	7.66E-01
32	1	175.41	4.86E-40	2	46.89	6.57E-11			
33	1	16.54	4.77E-05	2	5.10	7.81E-02	3	1.28	7.35E-01
37	4	223.57	3.20E-47	8	37.05	1.13E-05	8	16.73	3.30E-02
38	2	3.00	2.23E-01	3	4.81	1.86E-01	3	0.55	9.09E-01
47	1	205.53	1.30E-46	2	2.88	2.37E-01	3	0.25	9.69E-01
57	3	79.86	3.29E-17	6	51.41	2.45E-09	7	12.45	8.67E-02
58	1	57.35	3.64E-14	2	20.96	2.81E-05	2	12.21	2.23E-03
60	2	54.32	1.60E-12	4	36.71	2.07E-07	4	11.70	1.97E-02
67	2	158.66	3.53E-35	4	67.55	7.47E-14	6	2.20	9.01E-01
70	2	51.45	6.71E-12	4	91.99	4.97E-19	4	14.59	5.62E-03
71	3	218.36	4.54E-47	6	38.29	9.87E-07	6	0.43	9.99E-01
73	1	105.10	1.16E-24	2	10.82	4.47E-03			
74	4	413.58	3.23E-88	**7**	**107.55**	**2.96E-20**			
77	1	357.83	8.37E-80	2	66.49	3.65E-15			
78	1	68.57	1.23E-16	1	8.28	4.00E-03	1	0.59	4.42E-01
85	1	1.64	2.00E-01	2	0.61	7.39E-01	2	0.39	8.23E-01
86	1	249.70	3.02E-56	2	86.25	1.87E-19	**5**	**26.34**	**7.67E-05**
90	1	106.13	6.91E-25	1	3.00	8.32E-02	4	1.54	8.19E-01
96	3	120.33	6.56E-26	6	5.97	4.27E-01	7	4.12	7.66E-01
98	1	88.10	6.24E-21	2	0.14	9.30E-01	3	1.83	6.09E-01
104	1	88.10	6.24E-21	2	0.14	9.30E-01	3	1.83	6.09E-01
120	1	17.53	2.82E-05	2	6.12	4.68E-02	3	1.80	6.14E-01
133	1	38.62	5.15E-10	2	0.12	9.40E-01	2	0.87	6.49E-01
140	1	183.89	6.86E-42	2	11.17	3.75E-03	3	3.22	3.59E-01
142	1	38.62	5.15E-10	2	0.12	9.40E-01	2	0.87	6.49E-01
149	1	250.20	2.35E-56	2	2.18	3.36E-01	3	0.43	9.34E-01
180	2	52.65	3.69E-12	3	18.67	3.19E-04	4	5.81	2.13E-01
181	2	107.32	4.95E-24	2	23.39	8.35E-06	3	5.56	1.35E-01
189	2	33.94	4.28E-08	4	33.67	8.72E-07	5	14.02	1.55E-02
231	2	33.96	4.23E-08	3	34.11	1.88E-07	6	14.52	2.43E-02
233	2	331.00	1.33E-72	4	43.37	8.67E-09	8	14.59	6.77E-02
HLA-DQA1 amino acids									
-16	1	142.94	6.07E-33	1	0.22	6.41E-01	4	1.57	8.15E-01
11	1	106.13	6.91E-25	1	3.00	8.32E-02	4	1.54	8.19E-01
18	1	106.09	7.06E-25	1	3.00	8.33E-02	4	1.54	8.20E-01
25	1	94.70	2.21E-22	1	8.02	4.63E-03	4	0.21	9.95E-01
26	1	15.15	9.92E-05	1	8.20	4.20E-03	5	1.56	9.07E-01
34	1	121.86	2.48E-28	1	3.53	6.04E-02	4	2.72	6.06E-01
40	1	345.32	4.44E-77	1	9.97	1.59E-03			
41	1	15.77	7.15E-05	1	0.06	8.07E-01			
45	1	106.09	7.06E-25	1	3.00	8.33E-02	4	1.54	8.20E-01
47	3	361.58	4.63E-78	3	29.33	1.91E-06	10	1.56	9.99E-01
48	1	106.09	7.06E-25	1	3.00	8.33E-02	4	1.54	8.20E-01
50	2	346.31	6.32E-76	2	11.03	4.02E-03	7	1.55	9.80E-01
51	1	345.32	4.44E-77	1	9.97	1.59E-03			
52	2	248.76	9.62E-55	2	14.67	6.52E-04	7	1.55	9.81E-01
53	2	346.31	6.32E-76	2	11.03	4.02E-03	7	1.55	9.80E-01
54	1	77.97	1.05E-18	1	14.59	1.33E-04			
55	1	106.09	7.06E-25	1	3.00	8.33E-02	4	1.54	8.20E-01
56	2	161.14	1.02E-35	2	27.78	9.28E-07	9	1.56	9.97E-01
61	1	106.09	7.06E-25	1	3.00	8.33E-02	4	1.54	8.20E-01
64	1	106.09	7.06E-25	1	3.00	8.33E-02	4	1.54	8.20E-01
66	1	106.09	7.06E-25	1	3.00	8.33E-02	4	1.54	8.20E-01
69	2	151.71	1.14E-33	2	11.14	3.80E-03	7	1.57	9.80E-01
**75**	**1**	**424.82**	**2.18E-94**						
76	2	161.14	1.02E-35	2	27.78	9.28E-07	9	1.56	9.97E-01
80	1	106.09	7.06E-25	1	3.00	8.33E-02	4	1.54	8.20E-01
**107**	**1**	**424.96**	**2.03E-94**						
129	1	75.37	3.90E-18	1	2.47	1.16E-01	3	1.14	7.66E-01
130	1	15.79	7.06E-05	1	0.06	8.07E-01			
**156**	**1**	**424.96**	**2.03E-94**						
**161**	**1**	**424.96**	**2.03E-94**						
**163**	**1**	**424.96**	**2.03E-94**						
175	2	427.61	1.40E-93	1	3.07	7.99E-02	4	1.56	8.17E-01
187	1	15.16	9.86E-05	1	8.20	4.18E-03	5	1.56	9.06E-01
207	1	54.11	1.90E-13	1	0.71	3.99E-01	2	2.70	2.59E-01
215	1	90.67	1.69E-21	1	0.22	6.36E-01	4	1.55	8.17E-01
218	1	106.13	6.91E-25	1	3.00	8.32E-02	4	1.54	8.19E-01

Supplementary Table 9. Effects of individual amino acid residues at positions 75 of *HLA-DQA1* and 74 of the *HLA-DRB1* in Europeans.

Amino acid residues		Freq. Cases	Freq. Controls	OR	95% CI
HLA-DQA1 position 75	Ser	0.53	0.30	2.88	2.60-3.19
	Ile	0.30	0.53	0.35	0.31-0.38
HLA-DRB1 position 74	Arg	0.21	0.12	2.86	2.54-3.23
	Glu	0.07	0.06	1.17	0.97-1.40
	Ala	0.62	0.66	1.00	Reference
	Gln	0.08	0.13	0.62	0.53-0.73
	Leu	0.02	0.03	0.47	0.34-0.66

Supplementary Table 10. Haplotypes defined by HLA-DQA1 position 75 and HLA-DRB1 position 74 in Europeans. There were six European haplotypes defined by these two amino acid positions. The frequencies in cases and controls for each haplotype and the corresponding four-digit DRB1 and DQA1 classical alleles are provided. For derivation of effect estimates, we chose as reference one of the haplotypes with similar frequencies in cases and controls.

Amino acid					Frequency		Classical HLA alleles	
HLA-DQA1 **pos. 75**	***HLA-DRB1*** **pos. 74**	**OR**	**95%CI**	**P-value**	**Cases**	**Controls**	**DQA1**	**DRB1**
Ser	Arg	2.45	2.00-3.01	5.4E-79	0.31	0.11	**05:01*	**03:01*
Ser	Ala	1.35	1.01-1.65	2.3E-11	0.20	0.16	**05:01*	**12:01, *13:03*
Ile	Gln	0.53	0.42-0.67	2.3E-11	0.07	0.13	**02:01*	**07:01*
Ile	Leu	0.41	0.28-0.58	3.5E-08	0.01	0.03	**04:01*	*Other*
Ile	Glu	1.00	*Reference*	NA	0.06	0.06	**01:01, *0301*	*Other*
Ile	Ala	0.71	0.59-0.85	3.2E-37	0.35	0.50	**01:01, *01:02, *01:03, *03:01*	**01:01, *13:01, *13:02, *15:01*

Supplementary Table 11. Stepwise conditional analyses of all classical 4-digit alleles in HLA-DRB1 and HLA-DQA1 across all available cohorts combined.

Classical HLA-DQA1 and DRB1 alleles (4-digit resolution)	Freq. Cases	Freq. Controls	Unconditioned			Conditioned on DRB1*03:01			Conditioned on DRB1*03:01 and DQA1*05:01			Conditioned on DRB1*03:01 and DRB1*15:01, and DQA1*05:01		
			OR	SE	P value	OR	SE	P value	OR	SE	P value	OR	SE	P value
*HLA-DRB1*01:01*	0.02	0.05	0.54	0.08	1.4E-13	0.65	0.09	5.9E-07	0.74	0.09	5.4E-04	0.81	0.09	2.0E-02
*HLA-DRB1*03:01*	0.15	0.06	**3.71**	**0.05**	**2.9E-127**									
*HLA-DRB1*07:01*	0.06	0.09	0.48	0.07	5.1E-27	0.60	0.07	1.1E-13	0.67	0.07	2.1E-08	0.75	0.07	6.2E-05
*HLA-DRB1*12:01*	0.03	0.04	0.92	0.11	4.7E-01	1.02	0.12	8.5E-01	0.65	0.12	4.4E-04	0.66	0.12	7.9E-04
*HLA-DRB1*13:01*	0.02	0.03	0.69	0.09	4.9E-05	0.86	0.10	1.1E-01	0.98	0.10	8.0E-01	1.09	0.10	4.0E-01
*HLA-DRB1*13:02*	0.03	0.05	0.59	0.10	3.0E-07	0.74	0.10	4.8E-03	0.83	0.11	7.0E-02	0.92	0.11	4.0E-01
*HLA-DRB1*15:01*	0.18	0.09	1.35	0.06	4.0E-08	1.67	0.06	3.8E-19	**1.94**	**0.06**	**4.7E-29**			
*HLA-DQA1*01:01*	0.11	0.12	0.81	0.06	1.4E-04	0.99	0.06	9.0E-01	1.16	0.06	1.1E-02	1.34	0.06	1.3E-06
*HLA-DQA1*01:02*	0.23	0.17	1.00	0.05	9.7E-01	1.25	0.05	3.3E-06	1.48	0.05	7.2E-15	0.93	0.08	3.3E-01
*HLA-DQA1*01:03*	0.07	0.10	0.71	0.07	9.5E-07	0.84	0.07	1.7E-02	0.95	0.07	4.8E-01	1.08	0.07	3.3E-01
*HLA-DQA1*02:01*	0.05	0.09	0.48	0.07	1.1E-27	0.59	0.07	6.4E-14	0.66	0.07	9.3E-09	0.74	0.07	3.9E-05
*HLA-DQA1*03:01*	0.14	0.27	0.58	0.05	1.0E-29	0.71	0.05	2.6E-12	0.79	0.05	4.6E-06	0.93	0.05	1.6E-01
*HLA-DQA1*04:01*	0.01	0.02	0.44	0.15	5.4E-08	0.53	0.16	5.3E-05	0.60	0.16	1.4E-03	0.67	0.16	1.0E-02
*HLA-DQA1*05:01*	0.35	0.19	2.66	0.04	6.1E-122	**1.80**	**0.05**	**1.1E-30**						

Supplementary Table 12. ANNOVAR annotation of the top significant non-HLA SNPs and their proxies (R2>0.2). The lead SNP at each locus is highlighted.

Locus	SNP	Chr.	Position (hg19)	Risk allele	Non-risk allele	Odds ratio	P discovery	R^2 with top SNP in GWAS	Annotation
PLA2R1	rs17831251	2	160914156	C	T	2.25	1.2E-69	1.00	Intronic (top SNP in GWAS)
PLA2R1	rs17241973	2	160918601	A	G	2.03	6.2E-68	0.93	Intronic (top SNP in FUN-LDA annotation)
PLA2R1	rs3749119	2	160919020	C	T	1.95	7.2E-55	0.78	5' UTR
PLA2R1	rs35771982	2	160885418	G	C	1.94	2.5E-66	0.69	His300Asp (non-risk [His] -> risk [Asp])
PLA2R1	rs3749117	2	160885442	T	C	1.94	3.4E-66	0.68	Met292Val (non-risk [Val] -> risk [Met])
PLA2R1	rs3828323	2	160808075	C	T	1.50	2.6E-27	0.21	Gly1106Ser (non-risk [Ser] -> risk [Gly])
PLA2R1	rs1511223	2	160802644	A	G	1.42	6.1E-19	0.20	3' UTR
NFKB1	rs230540	4	103496667	T	C	1.25	9.0E-12	1.00	Intronic (top SNP in GWAS)
NFKB1	rs2272676	4	103423326	T	G	1.26	4.6E-10	0.70	Splice-5
IRF4	rs9405192	6	382537	G	A	1.29	2.1E-10	1.00	Intergenic (top SNP in GWAS)
IRF4	rs872071	6	411064	G	A	1.17	3.5E-06	0.21	3' UTR
IRF4	rs1050976	6	408079	T	C	1.17	3.1E-06	0.21	3' UTR
IRF4	rs1050979	6	410417	G	A	1.17	3.8E-06	0.21	3' UTR
IRF4	rs9391997	6	409119	G	A	1.19	2.2E-06	0.20	3' UTR
IRF4	rs1050975	6	408012	A	G	1.22	2.5E-04	0.15	3' UTR

Supplementary Table 13. Pleiotropy analysis. All phenotypes associated with MN susceptibility alleles were identified based on the GWAS catalogue (accessed 4/18) by interrogating all SNPs with R2 > 0.2 with at least one of the genome-wide significant SNPs independently associated with MN.

Previously published GWAS associations									Associations with MN				Top SNP	LD with the top SNP
Locus	DISEASE/TRAIT	PMID	Journal	Year	Index SNP	Risk Allele	OR	P value	Risk allele	OR	P value	Direction	SNP	R2
2q24.2	Membranous nephropathy	21323541	N Engl J Med	2011	rs4664308	A	2.28	9.0E-29	A	2.02	4.2E-68	Concordant	rs17831251	0.98
2q24.2	Waist circumference	25673412	Nature	2015	rs2124969	T	1.02	7.0E-09	T	2.03	1.7E-67	Concordant	rs17831251	0.88
2q24.2	Membranous nephropathy	27333618	NDT	2016	rs17830558	T	1.87	4.0E-10	T	1.63	7.2E-40	Concordant	rs17831251	0.32
2q24.2	Inflammatory bowel disease	26192919	Nat Genet	2015	rs4664304	A	1.06	3.0E-08	A	1.53	3.5E-30	Concordant	rs17831251	0.24
4q24	Monocyte percentage	27863252	Cell	2016	rs11097789	C	1.02	1.0E-09	C	1.28	1.3E-11	Concordant	rs230540	1.00
4q24	Lymphocyte percentage	27863252	Cell	2016	rs4648045	C	1.04	2.0E-21	C	1.25	1.6E-10	Concordant	rs230540	0.97
4q24	Lymphocyte counts	27863252	Cell	2016	rs5026472	G	1.04	1.0E-31	G	1.17	3.6E-06	Concordant	rs230540	0.49
4q24	Ulcerative colitis	23128233	Nature	2012	rs3774959	A	1.12	4.0E-12	A	1.25	1.8E-10	Concordant	rs230540	0.97
4q24	Ulcerative colitis	26192919	Nat Genet	2015	rs3774937	C	1.10	5.0E-14	C	1.26	3.0E-10	Concordant	rs230540	0.70
4q24	Primary biliary cholangitis	21399635	Nat Genet	2011	rs7665090	G	1.26	4.0E-12	G	1.17	3.7E-06	Concordant	rs230540	0.50
4q24	Primary biliary cholangitis	26394269	Nat Commun	2015	rs1054037	T	1.22	8.0E-10	T	1.17	4.1E-06	Concordant	rs230540	0.50
4q24	Glomerular filtration rate	26831199	Nat Commun	2016	rs228611	A	0.01	4.0E-12	G	1.16	2.3E-05	Opposed	rs230540	0.47
4q24	Tonsillectomy	28928442	Nat Commun	2017	rs230523	T	1.07	5.0E-14	C	1.26	1.8E-10	Opposed	rs230540	0.94
4q24	Allergic disease	29083406	Nat Genet	2017	rs227275	C	1.03	4.0E-11	A	1.13	3.2E-04	Opposed	rs230540	0.41
6p25.3	Crohn's disease	26192919	Nat Genet	2015	rs7773324	A	1.08	1.0E-09	A	1.16	1.9E-05	Concordant	rs9405192	0.59
6p25.3	Inflammatory bowel disease	26192919	Nat Genet	2015	rs7773324	A	1.06	6.0E-09	A	1.16	1.9E-05	Concordant	rs9405192	0.59
6p25.3	Chronic lymphocytic leukemia	28165464	Nat Commun	2017	rs9392504	A	1.33	1.0E-28	A	1.18	2.4E-06	Concordant	rs9405192	0.22
6p25.3	Chronic lymphocytic leukemia	18758461	Nat Genet	2008	rs872071	G	1.54	2.0E-20	G	1.18	3.5E-06	Concordant	rs9405192	0.21
6p25.3	Chronic lymphocytic leukemia	23770605	Nat Genet	2013	rs872071	G	1.33	6.0E-20	G	1.18	3.5E-06	Concordant	rs9405192	0.21
6p25.3	Chronic lymphocytic leukemia	24292274	Nat Genet	2013	rs872071	G	1.39	3.0E-16	G	1.18	3.5E-06	Concordant	rs9405192	0.21
6p25.3	Chronic lymphocytic leukemia	22700719	Blood	2012	rs872071	G	1.47	8.0E-14	G	1.18	3.5E-06	Concordant	rs9405192	0.21
6p25.3	Chronic lymphocytic leukemia	26956414	Nat Commun	2016	rs9391997	G	1.35	9.0E-22	G	1.18	2.2E-06	Concordant	rs9405192	0.20
6p21.32	ASD or schizophrenia	28540026	Mol Autism	2017	rs142972412	T	1.09	1.00E-12	n.a.	n.a.	n.a.	Unknown	rs3135335	0.25
6p21.32	Autoimmune hepatitis type-1	24768677	Gastroenterology	2014	rs2187668	T	2.90	2.00E-78	n.a.	n.a.	n.a.	Concordant	rs9273325	0.29
6p21.32	Celiac disease	20190752	Nat Genet	2010	rs2187668	A	6.23	1.00E-50	n.a.	n.a.	n.a.	Concordant	rs9273325	0.29
6p21.32	Celiac disease	17558408	Nat Genet	2007	rs2187668	A	7.04	1.00E-19	n.a.	n.a.	n.a.	Concordant	rs9273325	0.29
6p21.32	Childhood ear infection	28928442	Nat Commun	2017	rs4329147	T	1.11	1.00E-11	n.a.	n.a.	n.a.	Concordant	rs3135335	0.33
6p21.32	Childhood ear infection	27182965	Nat Genet	2016	rs4329147	T	1.11	1.00E-11	n.a.	n.a.	n.a.	Concordant	rs3135335	0.33
6p21.32	Childhood ear infection	28928442	Nat Commun	2017	rs3129868	C	0.08	6.00E-10	C	1.36	2.2E-07	Concordant	rs3135335	0.30
6p21.32	Complement C3 and C4 levels	23028341	PLoS Genet	2012	rs3763317	C	0.12	9.00E-66	C	1.57	5.0E-35	Concordant	rs28383314	0.21
6p21.32	Crohn's disease	23850713	Gut	2013	rs9271366	A	1.66	5.00E-12	G	1.27	1.2E-05	Opposed	rs28383314	0.37
6p21.32	Cutaneous lupus erythematosus	25827949	Exp Dermatol	2015	rs2187668	T	2.93	4.00E-10	n.a.	n.a.	n.a.	Concordant	rs9273325	0.29
6p21.32	Drug-induced liver injury	21570397	Gastroenterology	2011	rs9274407	A	3.10	5.00E-14	n.a.	n.a.	n.a.	Opposed	rs3135335	0.24
6p21.32	Eosinophil counts	27863252	Cell	2016	rs28383314	C	0.06	1.00E-60	T	2.37	3.1E-117	Opposed	rs28383314	1.00
6p21.32	Eosinophil percentage	27863252	Cell	2016	rs28383314	C	0.04	7.00E-27	T	2.37	3.1E-117	Opposed	rs28383314	1.00
6p21.32	Epstein-Barr virus response	23326239	PLoS Genet	2013	rs2854275	T	0.45	2.00E-10	n.a.	n.a.	n.a.	Concordant	rs9273325	0.35
6p21.32	Frontotemporal dementia	24943344	Lancet Neurol	2014	rs9268856	C	1.24	6.00E-09	A	1.35	1.2E-13	Opposed	rs9273325	0.22
6p21.32	Hematocrit	27863252	Cell	2016	rs9273039	T	0.04	1.00E-25	n.a.	n.a.	n.a.	Concordant	rs9273325	0.43
6p21.32	Hepatitis B	28928442	Nat Commun	2017	rs9268652	G	1.32	3.00E-09	G	1.36	8.1E-10	Concordant	rs3135335	0.40
6p21.32	Hepatitis C induced cirrhosis	23321320	J Hepatol	2013	rs910049	A	1.46	9.00E-11	n.a.	n.a.	n.a.	Opposed	rs3135335	0.26
6p21.32	Hepatitis C induced cirrhosis	23321320	J Hepatol	2013	rs3129860	A	1.36	1.00E-09	G	1.36	2.2E-07	Opposed	rs3135335	0.30
6p21.32	Hodgkin's lymphoma	21037568	Nat Genet	2010	rs6903608	G	1.70	3.00E-50	C	1.52	3.5E-29	Concordant	rs28383314	0.25
6p21.32	Hodgkin's lymphoma	24920014	Nat Commun	2014	rs6903608	C	1.64	7.00E-31	C	1.52	3.5E-29	Concordant	rs28383314	0.25
6p21.32	Hodgkin's lymphoma	24149102	Nat Commun	2013	rs6903608	G	1.62	5.00E-27	C	1.52	3.5E-29	Concordant	rs28383314	0.25
6p21.32	Membranous nephropathy	21323541	N Engl J Med	2011	rs2187668	A	4.32	8.00E-93	n.a.	n.a.	n.a.	Concordant	rs9273325	0.29
6p21.32	Immunoglobulin A	20694011	Nat Genet	2010	rs2187668	A	2.53	2.00E-33	n.a.	n.a.	n.a.	Concordant	rs9273325	0.29
6p21.32	Immunoglobulin A	20694011	Nat Genet	2010	rs9271366	A	7.69	3.00E-33	G	1.27	1.2E-05	Opposed	rs28383314	0.37
6p21.32	Itch from mosquito bite	28199695	Hum Mol Genet	2017	rs3104376	T	0.05	2.00E-28	n.a.	n.a.	n.a.	Opposed	rs3135335	0.23
6p21.32	Membranous nephropathy	27333618	NDT	2016	rs9272729	A	7.07	2.00E-39	n.a.	n.a.	n.a.	Concordant	rs9273325	0.29
6p21.32	Multiple sclerosis	19525955	Nat Genet	2009	rs9271366	G	2.78	7.00E-184	G	1.27	1.2E-05	Concordant	rs28383314	0.37
6p21.32	Multiple sclerosis	20159113	Am J Hum Genet	2010	rs3135338	A	3.43	2.00E-25	n.a.	n.a.	n.a.	Concordant	rs3135335	1.00
6p21.32	Multiple sclerosis	20453840	Nat Genet	2010	rs2040406	G	2.05	1.00E-20	n.a.	n.a.	n.a.	Concordant	rs9273325	0.63
6p21.32	Multiple sclerosis	20598377	J Neuroimmunol	2010	rs9271366	G	2.62	4.00E-17	G	1.27	1.2E-05	Concordant	rs28383314	0.37
6p21.32	Multiple sclerosis (OCB status)	23472185	PLoS One	2013	rs3129871	C	1.72	6.00E-15	C	1.20	5.8E-06	Concordant	rs3135335	0.90
6p21.32	Nasopharyngeal carcinoma	20512145	Nat Genet	2010	rs28421666	A	1.49	2.00E-18	A	1.39	7.4E-04	Concordant	rs9273325	0.45
6p21.32	Nodular sclerosis Hodgkins	29196614	Nat Commun	2017	rs9269081	A	1.82	2.00E-39	A	1.56	7.6E-33	Concordant	rs28383314	0.28
6p21.32	Red blood cell count	27863252	Cell	2016	rs9272535	A	0.05	6.00E-30	n.a.	n.a.	n.a.	Unknow	rs9273325	0.21
6p21.32	Reticulocyte count	27863252	Cell	2016	rs9270493	C	0.06	1.00E-46	n.a.	n.a.	n.a.	Concordant	rs28383314	0.41
6p21.32	Shingles	28928442	Nat Commun	2017	rs9268557	T	1.10	4.00E-15	T	1.17	9.3E-06	Concordant	rs3135335	0.27
6p21.32	Sum eosinophil basophil counts	27863252	Cell	2016	rs28383314	C	0.06	9.00E-64	T	2.37	3.1E-117	Opposed	rs28383314	1.00
6p21.32	Systemic lupus erythematosus	21408207	PLoS Genet	2011	rs2187668	A	2.23	6.00E-28	n.a.	n.a.	n.a.	Concordant	rs9273325	0.29
6p21.32	Systemic lupus erythematosus	23273568	Am J Hum Genet	2012	rs9270984	T	1.73	5.00E-24	n.a.	n.a.	n.a.	Opposed	rs3135335	0.34
6p21.32	Systemic lupus erythematosus	18204098	N Engl J Med	2008	rs2187668	A	n.a.	3.00E-21	n.a.	n.a.	n.a.	Concordant	rs9273325	0.29
6p21.32	Systemic lupus erythematosus	26502338	Nat Genet	2015	rs9273076	T	1.30	8.00E-13	n.a.	n.a.	n.a.	Concordant	rs28383314	0.26
6p21.32	Systemic lupus erythematosus	26316170	Arthritis Rheumatol	2015	rs2187668	n.a.	n.a.	1.00E-09	n.a.	n.a.	n.a.	Unknow	rs9273325	0.29
6p21.32	Systemic lupus erythematosus	26316170	Arthritis Rheumatol	2015	rs3129716	n.a.	n.a.	4.00E-09	n.a.	n.a.	n.a.	Unknow	rs9273325	0.34
6p21.32	Systemic sclerosis	21779181	PLoS Genet	2011	rs3129763	A	1.65	1.00E-11	A	2.44	6.5E-107	Concordant	rs28383314	0.44
6p21.32	Tuberculosis	28928442	Nat Commun	2017	rs3135359	C	1.21	9.00E-21	C	1.18	1.7E-04	Concordant	rs3135335	0.71
6p21.32	Type 1 diabetes	19430480	Nat Genet	2009	rs9268645	n.a.	n.a.	1.00E-100	C	1.07	4.9E-02	Unknow	rs3135335	0.38
6p21.32	Type 1 diabetes	17632545	Nature	2007	rs2647044	A	8.30	1.00E-16	n.a.	n.a.	n.a.	Concordant	rs9273325	0.34
6p21.32	Inflammatory Bowel Disease	21699788	Gastroenterology	2011	rs9271366	G	4.44	2.00E-70	G	1.27	1.2E-05	Concordant	rs28383314	0.37
6p21.32	Vogt-Koyanagi-Harada syndrome	25108386	Nat Genet	2014	rs3021304	G	2.97	1.00E-118	C	1.51	1.9E-29	Opposed	rs3135335	0.23
6p21.32	Waist-to-hip ratio	28443625	Nat Commun	2017	rs7759742	A	0.02	1.00E-09	T	1.65	1.1E-42	Unknow	rs28383314	0.24
6p21.32	Waist-to-hip ratio	25673412	Nature	2015	rs7759742	A	0.02	2.00E-11	T	1.65	1.1E-42	Unknow	rs28383314	0.24
6p21.32	Waist-to-hip ratio	25673412	Nature	2015	rs7759742	A	0.02	4.00E-11	T	1.65	1.1E-42	Unknow	rs28383314	0.24

Supplementary Table 14. Annotation of the suggestive loci for novel podocyte antigen candidate genes. A total of 20 genes encoded by the suggestive loci were predicted to be podocyte-expressed with a probability greater than 0.5; in total, 11/20 of predicted genes were detected in a mouse podocyte proteome and 3/20 have been experimentally implicated in podocyte biology based on our PubMed literature review (highlighted in yellow). All suggestive loci were tested for genetic interactions with classical HLA alleles, and the top interacting allele is listed for each locus.

Chr.	Position (Hg19)	Index SNP	Genes within the locus	P (discovery)	P (replication)	P (combined)	Best Interacting HLA allele	P (interaction)	Potential podocyte gene	Probability of human podocyte mRNA expression	Mouse Podocyte Preteome (PMID:29791858)	PubMed Literature Searches
14	72636594	rs149665	*[RGS6]*	2.1E-07	2.8E-01	5.3E-07	HLA_DQA1_0102	3.1E-03	*RGS6*	0.67	Absent	NA
12	64224210	rs59823810	*[SRGAP1,TMEM5]*	6.7E-07	1.6E-01	8.2E-07	HLA_DQA1_0102	2.6E-09	*TMEM5*	0.73	Absent	NA
2	225294852	rs6721570	*[CUL3,FAM124B]*	1.3E-06	2.2E-01	1.4E-06	HLA_DQA1_0401	2.0E-02	*CUL3; FAM1248*	0.87; 0.75	Present; Absent	NA
5	115852960	rs6860340	*[SEMA6A]*	2.7E-05	1.9E-01	2.0E-05	HLA_DPA1_0202	2.0E-03	*SEMA6A*	0.78	Absent	NA
6	166771814	rs9348123	*[MPC1,PRR18,RPS6KA2,SFT2D1]*	4.1E-05	3.3E-01	2.8E-05	HLA_DRB1_1501	1.0E-06	*RPS6KA2*	0.83	Absent	NA
2	111817805	rs71431137	*[ACOXL]*	7.2E-06	7.4E-01	4.3E-05	HLA_DPB1_0301	4.9E-03	*ACOXL*	0.73	Absent	NA
19	50995880	rs4802709	*[ASPDH,EMC10,FAM71E1,JOSD2, LRRC4B,MYBPC2,SPIB]*	2.6E-07	5.1E-02	9.0E-05	HLA_DRB1_1501	2.4E-04	*SPIB; MYBPC2*	0.62; 0.62	Absent	NA
11	132602735	rs3018404	*[OPCML]*	3.4E-05	6.5E-01	1.1E-04	HLA_DRB1_1501	1.0E-10	*OPCML*	0.84	Absent	NA
15	80719445	rs72745632	*[ARNT2]*	2.7E-05	7.1E-01	1.4E-04	HLA_DQA1_0201	3.4E-03	*ARNT2*	0.84	Present	NA
12	16675898	rs4140827	*[LMO3]*	3.2E-05	9.0E-01	1.6E-04	HLA_DQA1_0102	1.0E-03	*LMO3*	0.85	Absent	NA
8	40306342	rs9298595	*[ZMAT4]*	2.5E-05	8.5E-01	2.3E-04	HLA_DRB1_1501	1.7E-02	*ZMAT4*	0.70	Absent	NA
6	110132871	rs4945839	*[FIG4]*	1.6E-05	6.8E-01	3.7E-04	HLA_DQA1_0401	8.1E-02	*FIG4**	0.76	Present	Podocyte PIKfyve complex (PMID: 28216340)
1	147281349	rs1692144	*[GJA5]*	3.8E-05	4.2E-01	4.7E-04	HLA_DQA1_0102	1.5E-03	*GJA5*	0.91	Absent	NA
7	1423966	rs139458785	*[INTS1,MAFK,MICALL2,PSMG3,T MEM184A]*	2.2E-05	3.6E-01	5.7E-04	HLA_DRB1_1301	2.8E-02	*MAFK; INTS1*	0.75; 0.62	Present; Present	NA
1	161716885	rs35801335	*[ATF6,DUSP12,FCRLB]*	2.1E-05	2.8E-01	5.9E-04	HLA_DRB1_1501	4.1E-03	*DUSP12; ATF6**	0.76; 0.62	Present; Absent	ATF6-dependent ER-stress response in podocytes (PMID: 25754093, 29500363)
3	59737251	rs780375	*[FHIT]*	4.7E-05	1.5E-01	9.5E-04	HLA_DRB1_1501	4.6E-08	*FHIT*	0.59	Present	NA
1	164638458	rs61803846	*[PBX1]*	3.5E-05	9.5E-01	1.2E-03	HLA_DRB1_1501	2.0E-04	*PBX1*	0.72	Present	NA
3	65379467	rs1016552	*[MAGI1]*	1.1E-05	5.6E-01	2.3E-03	HLA_DRB1_1501	1.3E-14	*MAGI1**	0.64	Present	MAGI1 directly interacts with nephrin (PMID: 27707879)
5	122376331	rs1437624	*[PPIC,SNX2,SNX24]*	4.1E-05	7.7E-02	1.5E-02	HLA_DRB1_1501	4.1E-10	*SNX24; SNX2*	0.80; 0.69	Present; Absent	NA
5	122368738	rs12055325	*[PPIC]*	4.7E-05	5.6E-02	2.0E-02	HLA_DRB1_1501	8.1E-10	*PPIC*	0.52	Present	NA

* literature supported genes

Supplementary Table 15. SNP-based genetic risk score (GRS) models. There were a total of six SNPs with independent contributions to MN risk and two statistically-significant interaction terms based on joint analyses of all cohorts. For East-Asian cohorts, there were five independent genome-wide significant SNPs and one significant interaction term. For Europeans, there were six independent SNPs and one significant interaction term. The provided mutually-adjusted effect sizes for those SNPs are derived from final GRS models that include significant interaction terms.

Joint				East Asian				European			
SNP	Effect	StdErr	P value	SNPs	Effect	StdErr	P value	SNPs	Effect	StdErr	P value
rs28383314 (HLA1)	0.54	0.10	9.8E-08	rs9269027 (HLA1)	0.69	0.21	1.2E-03	rs9271541 (HLA1)	0.35	0.12	4.0E-03
rs9273325 (HLA2)	0.38	0.13	2.8E-03	rs1974461 (HLA2)	1.24	0.22	9.8E-09	rs9265949 (HLA2)	0.68	0.08	6.5E-18
rs3135335 (HLA3)	0.34	2.00	4.1E-19	rs6707458 (PLA2R1)	0.37	0.13	5.0E-03	rs2858309 (HLA3)	0.31	0.05	3.4E-09
rs6707458 (PLA2R1)	0.43	0.07	2.1E-09	rs230540 (NFKB1)	0.25	0.07	4.3E-04	rs6707458 (PLA2R1)	0.35	0.07	3.7E-06
rs230540 (NFKB1)	0.26	0.04	3.0E-10	rs9405192 (IRF4)	0.39	0.07	5.0E-08	rs230540 (NFKB1)	0.17	0.05	6.9E-04
rs9405192 (IRF4)	0.28	0.04	6.4E-13	rs9269027:rs6707458	0.49	0.13	1.6E-04	rs9405192 (IRF4)	0.18	0.05	7.1E-04
rs28383314:rs6707458	0.17	0.06	1.0E-02					rs9271541:rs6707458	0.34	0.08	1.5E-05
rs9273325:rs6707458	0.20	0.08	1.2E-02								

Supplementary Table 16. Clinical correlations of genetic risk score (GRS). We tested the correlations between the ethnicity-specific GRS and five clinical traits from the time of kidney biopsy: age at diagnosis, 24-hour proteinuria, nephrotic range proteinuria, estimated GFR, serum albumin, and serum anti-PLA2R1 sero-positivity, and serum anti-PLA2R1 antibody titers among those with detectable levels; the statistics for each trait before and after adjustment for age, sex and ethnicity are provided.

		Minimally adjusted Model			Fully Adjusted Model		
	No. of cases	Effect size	SE	P	Effect size	SE	P
Age at biopsy	1752	-0.38	0.36	2.9E-01	-0.3	0.37	4.1E-01
eGFR	745	-0.004	0.02	8.1E-01	0.01	0.01	5.7E-01
Albumin	316	-0.05	0.02	2.3E-03	-0.01	0.02	6.0E-01
24-hour proteinuria level	1329	0.12	0.04	1.4E-03	0.12	0.04	1.3E-03
Nephrotic (proteinuria ≥ 3.5 g/d)	1329	0.14	0.05	8.3E-03	0.14	0.06	9.6E-03
Positive anti-PLA2R1 status	1114	0.34	0.06	2.6E-08	0.34	0.06	9.0E-08
Anti-PLA2R1 antibody titer	892	0.4	0.07	3.1E-09	0.42	0.07	1.2E-09

Supplementary Table 17. Diagnostic Properties of Serum Anti-PLA2R Antibody ELISA in GWAS Discovery and Validation Cohorts with Available Sera. The standard diagnostic cut-off of 20 units/mL was applied as recommended by the manufacturer. Sensitivity, Specificity, Positive Likelihood Ratio (LR+) and Negative Likelihood Ratio (LR-) were calculated for each discovery and validation cohorts individually and jointly. Only serum samples obtained within 6 months of the diagnostic biopsy were included in the analysis.

Cohort	Sample Size	Specificity	Sensitivity	LR+	LR-
East Asian Discovery	MN (N=304) vs. healthy controls (N=56)	100%	60%	Inf	0.40
European Discovery	MN (N=810) vs. healthy controls (N=99)	99%	57%	57.0	0.43
All Discovery	MN (N=1114) vs. healthy controls (N=197)	99%	58%	58.0	0.42
GWAS Validation	MN (N=248) vs. healthy controls (N=145)	100%	51%	Inf	0.49
NEPTUNE Validation	MN (N=36) vs. diseased controls (N=111)	99%	53%	53.0	0.47
All European Validation	MN (N=339) vs. all controls (N=256)	100%	51%	Inf	0.49

Supplementary Table 18. Diagnostic Performance of the Genetic Risk Score (GRS) and the Combined Risk Score (CRS) in (A) the East Asian Discovery Cohorts; (B) the European Discovery Cohorts; (C) European GWAS Validation Cohorts; (D) NEPTUNE European-American Validation Cohorts; (E) All Validation Cohorts Combined. The Genetic Risk Score (GRS) was calculated for all cases and healthy controls with available genetic data; to demonstrate disease specificity, additional ethnicity-matched diseased controls with a biopsy diagnosis of FSGS or IgAN were genotyped and included in the analysis of the discovery cohorts. The CRS was calculated for a subset of individuals with both serum measurements and genetic data (the exact numbers used in each analysis are indicated in parentheses).

A. East Asian Discovery Performance

GRS	AUROC (95%CI)	P	CRS	AUROC (95%CI)	P
MN (N=806) vs. controls (N=1970)	0.80 (0.78-0.82)	3.1E-114	MN (N=304) vs. controls (N=56)	0.93 (0.91-0.96)	4.2E-11
MN (N=806) vs. FSGS (N=52)	0.79 (0.73-0.85)	3.0E-11	MN (N=304) vs. FSGS (N=52)	0.99 (0.98-0.99)	1.1E-08
MN (N=806) vs. IGAN (N=47)	0.79 (0.74-0.85)	7.2E-11	MN (N=304) vs. IGAN (N=47)	0.97 (0.96-0.99)	2.9E-09
MN (N=806) vs. ALL (N=2069)	0.80 (0.78-0.82)	6.4E-117	MN (N=304) vs. ALL (N=155)	0.96 (0.95-0.98)	4.3E-18

B. European Discovery Performance

GRS	AUROC (95%CI)	P	CRS	AUROC (95%CI)	P
MN (N=1910) vs. controls (N=2676)	0.75 (0.74-0.77)	4.3E-162	MN (N=810) vs. controls (N=99)	0.87 (0.84-0.90)	9.4E-24
MN (N=1910) vs. FSGS (N=37)	0.72 (0.64-0.80)	2.0E-05	MN (N=810) vs. FSGS (N=37)	0.91 (0.86-0.96)	1.1E-12
MN (N=1910) vs. IGAN (N=88)	0.78 (0.73-0.82)	3.2E-15	MN (N=810) vs. IgAN (N=88)	0.90 (0.88-0.93)	1.9E-25
MN (N=1910) vs. ALL (N=2801)	0.75 (0.74-0.77)	4.3E-166	MN (N=810) vs. ALL (N=224)	0.89 (0.87-0.91)	1.2E-46

C. European GWAS Validation Datasets

GRS	AUROC (95%CI)	P	CRS	AUROC (95%CI)	P
UK Cohort: MN (N=333) vs. controls (N=348)	0.78 (0.75-0.82)	5.9E-30	MN (N=176) vs. controls (N=145)	0.96 (0.94-0.98)	7.3E-19
Dutch Cohort: MN (N=142) vs. controls (N=1832)	0.75 (0.71-0.80)	7.6E-29	MN (N=72) vs. controls (N=145)	0.99 (0.98-1.00)	2.0E-07
French Cohort: MN (N=75) vs. controls (N=157)	0.74 (0.66-0.81)	4.3E-09	MN (N=75) vs. controls (N=157)*	NA	NA
Combined: MN (N=550) vs. controls (N=2337)	0.78 (0.75-0.80)	1.5E-84	MN (N=303) vs. controls (N=145)	0.97 (0.95-0.98)	6.9E-21

** serum ELISA test not available for this cohort*

D. NEPTUNE European-American Validation

GRS	AUROC (95%CI)	P	CRS	AUROC (95%CI)	P
MN (N=46) vs. FSGS (N=62)	0.71 (0.61-0.81)	1.9E-04	MN (N=36) vs. FSGS (N=53)	0.89 (0.81-0.97)	3.8E-07
MN (N=46) vs. MCD (N=58)	0.68 (0.58-0.78)	1.1E-03	MN (N=36) vs. MCD (N=44)	0.88 (0.80=0.96)	1.3E-06
MN (N=46) vs. IgAN (N=14)	0.75 (0.62-0.89)	4.7E-03	MN (N=36) vs. IgAN (N=14)	0.91 (0.83-0.99)	2.0E-04
MN (N=46) vs. All (N=134)	0.70 (0.61-0.79)	1.5E-05	MN (N=36) vs. All (N=111)	0.89 (0.81-0.96)	3.8E-09

E. All Validation Cohorts Combined

GRS	AUROC (95%CI)	P	CRS	AUROC (95%CI)	P
MN (N=596) vs. all controls (N=2471)	0.77 (0.75-0.79)	1.72E-88	MN (N=339) vs. all controls (N=256)	0.96 (0.94-0.97)	1.48E-31

Supplementary Table 19. Diagnostic Properties of Serum, Genetic, and Combined Tests for a Range of Diagnostic Cut-offs: these tables refer to the four clinical scenarios (A-D) discussed in the Supplementary Results; sensitivity, specificity, disease likelihood ratio for the positive test (LR+) and negative test (LR-) are calculated for each cut-off based on all available cohorts (the exact numbers of cases and controls used are provided in brackets).

A. Clinical Scenario 1. Only serum test available: diagnostic properties of the serum PLA2R antibody ELISA for test cut-offs of 20, 14, and 2 U/mL by ethnicity; shaded row corresponds to the standard diagnostic cut-off of 20 U/mL.

Serum Test East Asians (304 cases and 155 controls)				
**Threshold**	**Specificity**	**Sensitivity**	**LR+**	**LR-**
20 U/mL	100%	60%	Inf	0.40
2 U/mL	92%	83%	10.4	0.18

Serum Test Europeans (1,094 cases and 480 controls)				
**Threshold**	**Specificity**	**Sensitivity**	**LR+**	**LR-**
20 U/mL	100%	57%	Inf	0.43
2 U/mL	73%	75%	2.8	0.34

B. Clinical Scenario 2. Both serum and genetic tests available: diagnostic properties of the Combined Risk Score (CRS) for the cut-offs in the high specificity range (from 95% to 100%) by ethnicity; shaded row corresponds to the specific example discussed under the Clinical Scenario 2.

CRS: East Asians (304 cases and 155 controls)				
**Threshold**	**Specificity**	**Sensitivity**	**LR+**	**LR-**
1.58	100%	73%	Inf	0.27
1.45	99%	74%	74.0	0.26
1.15	98%	79%	39.5	0.21
1.00	97%	82%	27.3	0.19
0.86	96%	85%	21.3	0.16
0.84	95%	85%	17.0	0.16

CRS: European Cohorts (1,094 cases and 480 controls)				
**Threshold**	**Specificity**	**Sensitivity**	**LR+**	**LR-**
3.00	100%	49%	Inf	0.51
2.50	99%	60%	60.0	0.40
2.28	98%	64%	32.0	0.37
2.14	97%	66%	22.0	0.35
2.05	96%	67%	16.8	0.34
2.00	95%	67%	13.4	0.35

C. Clinical Scenario 3. Serum test is performed first and is negative: diagnostic properties of the Combined Risk Score (CRS) for the cut-offs in the high specificity range (from 95% to 100%) in the PLA2R Ab < 20 U/mL MN subgroup by ethnicity; shaded row corresponds to the example discussed under the Clinical Scenario 3.

CRS: PLA2R Ab- East Asians (123 cases and 155 controls)				
**Threshold**	**Specificity**	**Sensitivity**	**LR+**	**LR-**
1.58	100%	34%	Inf	0.66
1.45	99%	37%	37.0	0.64
1.15	98%	48%	24.0	0.53
0.98	97%	56%	18.7	0.45
0.86	96%	62%	15.5	0.40
0.84	95%	62%	12.4	0.40

CRS: PLA2R Ab- Europeans (473 cases and 480 controls)				
**Threshold**	**Specificity**	**Sensitivity**	**LR+**	**LR-**
3.00	100%	11%	Inf	0.89
2.50	99%	20%	20.0	0.81
2.28	98%	25%	12.5	0.77
2.14	97%	27%	9.0	0.75
2.05	96%	28%	7.0	0.75
2.00	95%	29%	5.8	0.75

D. Clinical Scenario 4. No serum test available: diagnostic properties of the Genetic Risk Score (GRS) for the cut-offs in the high specificity range (from 95% to 100%) by ethnicity; shaded row corresponds to the example discussed under the Clinical Scenario 4.

GRS: East Asians (806 cases and 2,070 controls)				
**Threshold**	**Specificity**	**Sensitivity**	**LR+**	**LR-**
3.20	100%	6%	Inf	0.94
2.78	99%	15%	15.0	0.86
2.58	98%	20%	10.0	0.82
2.00	97%	28%	9.3	0.74
1.75	96%	31%	7.8	0.72
1.69	95%	35%	7.0	0.68

GRS: Europeans (2,506 cases and 5,272 controls)				
**Threshold**	**Specificity**	**Sensitivity**	**LR+**	**LR-**
3.32	100%	7%	Inf	0.93
2.64	99%	13%	13.0	0.88
2.30	98%	18%	9.0	0.84
2.12	97%	21%	7.0	0.81
2.05	96%	26%	6.5	0.77
1.92	95%	28%	5.6	0.76

Supplementary Table 20. Extension of the NEPTUNE Validation Studies to All Ancestries: performance of the Genetic Risk Score (GRS) and the Combined Risk Score (CRS) in (A) NEPTUNE participants of all ancestries; (B) NEPTUNE participants of European-American ancestry; (C) NEPTUNE participants of African-American ancestry and (D) NEPTUNE participants of Hispanic-American ancestry. The genetic ancestry was defined by co-clustering with the reference populations from the 1000 Genomes Project.

A. NEPTUNE All Ancestries (N=475)

GRS	AUROC (95%CI)	P	CRS	AUROC (95%CI)	P
MN (N=89) vs. FSGS (N=184)	0.70 (0.63-0.77)	8.3E-08	MN (N=71) vs. FSGS (N=153)	0.85 (0.79-0.90)	8.7E-07
MN (N=89) vs. MCD (N=164)	0.71 (0.64-0.78)	9.7E-08	MN (N=71) vs. MCD (N=130)	0.85 (0.89-0.91)	1.1E-07
MN (N=89) vs. IgAN (N=38)	0.81 (0.73-0.88)	1.7E-06	MN (N=71) vs. IgAN (N=36)	0.90 (0.84-0.96)	6.4E-05
MN (N=89) vs. ALL (N=386)	0.71 (0.66-0.77)	1.4E-10	MN (N=71) vs. ALL (N=319)	0.85 (0.80-0.91)	1.1E-10

B. NEPTUNE European-American Ancestry (N=180)

GRS	AUROC (95%CI)	P	CRS	AUROC (95%CI)	P
MN (N=46) vs. FSGS (N=62)	0.71 (0.61-0.81)	1.9E-04	MN (N=36) vs. FSGS (N=53)	0.89 (0.81-0.97)	3.8E-07
MN (N=46) vs. MCD (N=58)	0.68 (0.58-0.78)	1.1E-03	MN (N=36) vs. MCD (N=44)	0.88 (0.80-0.96)	1.3E-06
MN (N=46) vs. IgAN (N=14)	0.75 (0.62-0.89)	4.7E-03	MN (N=36) vs. IgAN (N=14)	0.91 (0.83-0.99)	2.0E-04
MN (N=46) vs. ALL (N=134)	0.70 (0.61-0.79)	1.5E-05	MN (N=36) vs. ALL (N=111)	0.89 (0.81-0.96)	3.8E-09

C. NEPTUNE African-American Ancestry (N=133)

GRS	AUROC (95%CI)	P	CRS	AUROC (95%CI)	P
MN (N=18) vs. FSGS (N=70)	0.74 (0.62-0.87)	1.5E-03	MN (N=16) vs. FSGS (N=55)	0.86 (0.76-0.97)	1.0E-02
MN (N=18) vs. MCD (N=43)	0.76 (0.62-0.90)	3.7E-03	MN (N=16) vs. MCD (N=30)	0.86 (0.74-0.98)	1.4E-02
MN (N=18) vs. IgAN (N=2)	0.85 (0.67-1.00)	1.8E-01	MN (N=16) vs. IgAN (N=2)	0.92 (0.80-1.00)	2.2E-01
MN (N=18) vs. ALL (N=115)	0.75 (0.63-0.87)	6.5E-04	MN (N=16) vs. ALL (N=87)	0.86 (0.76-0.97)	3.0E-03

D. NEPTUNE Hispanic-Americans (N=94)

GRS	AUROC (95%CI)	P	CRS	AUROC (95%CI)	P
MN (N=18) vs. FSGS (N=34)	0.65 (0.50-0.80)	1.5E-01	MN (N=12) vs. FSGS (N=29)	0.70 (0.52-0.88)	6.8E-02
MN (N=18) vs. MCD (N=33)	0.62 (0.46-0.78)	3.0E-01	MN (N=12) vs. MCD (N=29)	0.70 (0.52-0.89)	5.9E-02
MN (N=18) vs. IgAN (N=9)	0.76 (0.54-0.97)	4.6E-02	MN (N=12) vs. IgAN (N=7)	0.86 (0.66-1.00)	1.1E-01
MN (N=18) vs. ALL (N=76)	0.65 (0.53-0.78)	1.1E-01	MN (N=12) vs. ALL (N=65)	0.72 (0.56-0.88)	1.9E-02

Supplementary Table 21. The Performance of the European, Trans-ethnic, and Asian Genetic Risk Scores (GRS) in Multiethnic NEPTUNE Validation Cohorts. (A) NEPTUNE participants of all ancestries; (B) NEPTUNE participants of African-American ancestry and (C) NEPTUNE participants of Hispanic-American ancestry. The genetic ancestry was defined by co-clustering with the reference populations from the 1000 Genomes Project.

A. NEPTUNE All Ancestries

NEPTUNE All Ancestries (N=475)	European-specific GRS		Trans-ethnic GRS		Asian-specific GRS	
	AUROC (95%CI)	P	AUROC (95%CI)	P	AUROC (95%CI)	P
MN (N=89) vs. FSGS (N=184)	0.70 (0.63-0.77)	8.3E-08	0.68 (0.61-0.75)	2.7E-06	0.52 (0.45-0.59)	4.7E-01
MN (N=89) vs. MCD (N=164)	0.71 (0.64-0.78)	9.7E-08	0.70 (0.63-0.77)	9.6E-07	0.53 (0.46-0.60)	6.1E-01
MN (N=89) vs. IgAN (N=38)	0.81 (0.73-0.88)	1.7E-06	0.78 (0.69-0.86)	5.4E-06	0.62 (0.51-0.72)	3.4E-02
MN (N=89) vs. ALL (N=386)	0.71 (0.66-0.77)	1.4E-10	0.70 (0.64-0.76)	2.0E-08	0.52 (0.45-0.58)	8.6E-01

B. NEPTUNE African-American Ancestry

NEPTUNE African-American Ancestry (N=133)	European-specific GRS		Trans-ethnic GRS		Asian-specific GRS	
	AUROC (95%CI)	P	AUROC (95%CI)	P	AUROC (95%CI)	P
MN (N=18) vs. FSGS (N=70)	0.74 (0.62-0.87)	1.5E-03	0.73 (0.60-0.85)	5.7E-03	0.51 (0.36-0.65)	9.0E-01
MN (N=18) vs. MCD (N=43)	0.76 (0.62-0.90)	3.7E-03	0.77 (0.64-0.91)	1.8E-03	0.59 (0.42-0.76)	2.9E-01
MN (N=18) vs. IgAN (N=2)	0.85 (0.67-1.00)	1.8E-01	0.88 (0.72-1.00)	1.1E-01	0.65 (0.39-0.92)	5.1E-01
MN (N=18) vs. ALL (N=115)	0.75 (0.63-0.87)	6.5E-04	0.75 (0.63-0.86)	1.5E-03	0.53 (0.38-0.67)	7.7E-01

C. NEPTUNE Hispanic-Americans

NEPTUNE Hispanic-American Ancestry (N=94)	European-specific GRS		Trans-ethnic GRS		Asian-specific GRS	
	AUROC (95%CI)	P	AUROC (95%CI)	P	AUROC (95%CI)	P
MN (N=18) vs. FSGS (N=34)	0.65 (0.50-0.80)	1.5E-01	0.38 (0.23-0.54)	3.5E-01	0.43 (0.26-0.60)	5.3E-01
MN (N=18) vs. MCD (N=33)	0.62 (0.46-0.78)	3.0E-01	0.59 (0.43-0.75)	4.3E-01	0.57 (0.40-0.73)	5.0E-01
MN (N=18) vs. IgAN (N=9)	0.76 (0.54-0.97)	4.6E-02	0.74 (0.53-0.95)	4.9E-02	0.81 (0.63-0.99)	3.0E-02
MN (N=18) vs. ALL (N=76)	0.65 (0.53-0.78)	1.1E-01	0.62 (0.50-0.74)	2.5E-01	0.60 (0.45-0.74)	2.8E-01

Supplementary Table 22: Population Frequencies of Individual Risk Alleles Contributing to the Genetic Risk Score (GRS). The frequency of risk aleles was estimated using three continental populations of the 1000 Genomes Project.

	Locus	Top SNP	Risk allele	East Asian Freq.	European Freq.	African Freq.
**Non-HLA (Common)**	***PLA2R1***	rs17831251	C	0.66	0.59	0.97
	NFKB1	rs230540	C	0.45	0.35	0.03
	IRF4	rs9405192	G	0.42	0.68	0.79
**HLA (European GRS)**	***HLA1***	rs9271541	C	0.12	0.22	0.34
	HLA2	rs9265949	T	0.004	0.08	0.02
	HLA3	rs2858309	C	0.19	0.34	0.32
**HLA (East Asian GRS)**	***HLA1***	rs9269027	A	0.35	0.28	0.31
	HLA2	rs1974461	T	0.02	0.02	0.06

SUPPLEMENTARY NOTE 1:

Clinical Implications of Genetic Risk Score (GRS):

Below, we consider four clinical scenarios that highlight the clinical utility of the GRS and its potential complementary use with the serum test to establish a diagnosis of MN.

Clinical Scenario 1. Only serum test available.

In this study, we analyzed serology data obtained within 6 months of a diagnostic biopsy for a total of 2,342 individuals (1,492 cases, 300 healthy controls, and 550 disease controls). These data represent the largest and most comprehensive evaluation of the serum PLA2R antibody test across diverse cohorts and clinical practice settings. In East Asians, based on the analysis of 304 cases, 56 healthy, and 99 disease controls, we estimate that the standard ELISA cut-off of 20 U/mL provides 100% specificity and 60% sensitivity for the diagnosis of MN. In European analysis of 1,094 cases, 244 healthy, and 236 diseased controls, the same cut-off provides 100% specificity and 57% sensitivity. This demonstrates that although PLA2R Ab test is highly specific, it is diagnostic in only 57-60% of cases across different clinical settings. The diagnostic properties of the serum test at cut-offs 20 and 2 U/mL are provided in **Supplementary Table 19A**. We note that the antibody level of 20 U/mL represents the standard diagnostic cut-off that is recommended by the ELISA manufacturer; levels 2-20 U/mL are considered as borderline-negative, and levels <2 U/mL are generally considered as negative[2].

Clinical Scenario 2. Both serum and genetic tests are available.

When both serum and genetic results are available, the Combined Risk Score (CRS) maximizes the diagnostic information between an inherited risk (GRS) and an antibody level, resulting in improved sensitivity compared to the serum test alone. For example, in East Asians, the standardized CRS cut-off of 1.00 (i.e. one standard deviation from the control mean) has 97% specificity and 82% specificity, with positive likelihood ratio of 27. Increasing the cut-off to 1.45 can establish the diagnosis of MN with 99% specificity and 74% sensitivity, with positive likelihood ratio of 74. For Europeans, the standardized CRS cut-off of 2.5 can establish the diagnosis of MN with 99% specificity, 60% sensitivity, and positive likelihood ratio of 60. The diagnostic properties of the CRS test at various cut-offs in the high specificity range (95%-100%) are summarized by ethnicity in **Supplementary Table 19B**.

Clinical Scenario 3. PLA2R Ab test is performed first and is negative (<20 U/mL).

Because the CRS uses genetic information to improve case classification even in the setting of low antibody titers, the genetic test may also be performed if the serum test is negative or inconclusive. The same CRS cut-offs as above can be used in this subgroup, but the sensitivity of the test is expected to be lower. For example, in East Asians the standardized CRS cut-off of 1.45 can establish the diagnosis of MN with 99% specificity, 37% sensitivity, and positive

likelihood ratio of 37. For Europeans, the CRS cut-off of 2.50 can establish the diagnosis of MN with 99% specificity, 20% sensitivity, and positive likelihood ratio of 20. This means that 20-37% of cases in the PLA2Ab negative subgroup could be potentially reclassified with very high confidence by adding the genetic test to their evaluation. Relaxing the cut-off to achieve the specificity of 97% results in 27% case classifications in Europeans (positive likelihood ratio of 9) and 56% in East Asians (positive likelihood ratio of over 18). The diagnostic properties of the CRS test in this particular clinical setting are provided in **Supplementary Table 19C**.

Clinical Scenario 4. No serum test available.

In some clinical settings, serum PLA2R Ab testing is not routinely available to clinicians. In this situation, the Genetic Risk Score (GRS) alone can be used to make the diagnosis of primary MN. In East Asians, based the analysis of 806 cases, 1,970 healthy, and 100 disease controls, the GRS 2.78 standard deviations from the control mean has 99% specificity, 15% sensitivity, and 15-fold positive likelihood ratio. In Europeans, based on the combined analysis of 2,506 cases, 5,013 healthy, and 259 disease controls, the standardized GRS cut-off 2.64 is diagnostic of MN with 99% specificity, 13% sensitivity, and 13-fold positive likelihood ratio. The sensitivity is relatively low for 99% specificity, but this means that 13-15% of cases can be diagnosed with primary MN with high level of certainty without the need for a serum test or a kidney biopsy. Notably, selecting a test cut-off that relaxes the specificity to 95% can identify up to one third of MN cases with approximately 7-fold positive likelihood ratio. The summary of diagnostic properties of the GRS test by ethnicity and at various diagnostic cut-offs is provided in **Supplementary Table 19D**.

SUPPLEMENTARY NOTE 2:

Supplementary Acknowledgments

The UK AUTO-MN Network:

We thank Jean Winterbottom for organizing the network and all nephrologists and MN patients from the following renal centers across UK who participated in the UK MRC AUTO-MN sample collection: Belfast City Hospital, Belfast; Bradford Teaching Hospitals NHS Trust, Bradford; Cambridge University Hospitals NHS Trust, Cambridge; Central Lancashire NHS Trust, Preston; Derriford Hospital, Plymouth; Dorset County NHS Foundation Trust, Dorchester; Edinburgh Royal Infirmary, Edinburgh; Freeman Hospital, Newcastle; Glasgow Western Infirmary, Glasgow; Guys and St Thomas Hospital, London; Hammersmith Hospital, London; Hull & East Yorkshire Hospitals Trust, Hull; Kings College Hospital Foundation Trust, London; Leicester General Hospital, Leicester; Lister Hospital, Stevenage; Manchester University Foundation Trust, Manchester, NHS Tayside Ninewells Hospital, Dundee, Northern General Hospital, Sheffield; Nottingham City Hospital, Nottingham; Oxford John Radcliffe Hospital, Oxford; Queen Alexandra Hospital, Portsmouth; Royal Cornwall Hospitals NHS Trust, Truro; Royal Devon & Exeter NHS Trust, Exeter; Royal Free Hospital, London; Royal Liverpool University Hospital, Liverpool; Salford Royal Foundation Trust, Salford; Shrewsbury & Telford NHS Trust, Shrewsbury; St Barts and the London Hospital, London; St George's Hospital, London; St James University Hospital, Leeds; University Hospital of North Staffs, Stoke; University Hospital of Wales, Cardiff; Western Health & Social Care Trust, Londonderry; York Teaching Hospital NHS Trust, York.

German Chronic Kidney Disease (GCKD) Study:

We thank all nephrologists who provide routine care for the patients and collaborate with the German Chronic Kidney Disease (GCKD) study. The list of the key GCKD Instigators includes: University of Erlangen-Nürnberg: Kai-Uwe Eckardt, Heike Meiselbach, Markus Schneider, Thomas Dienemann, Hans-Ulrich Prokosch, Barbara Bärthlein, Andreas Beck, Thomas Ganslandt, André Reis, Arif B. Ekici, Susanne Avendaño, Dinah Becker-Grosspitsch, Ulrike Alberth-Schmidt, Birgit Hausknecht, Rita Zitzmann, Anke Weigel; University of Freiburg: Anna Köttgen, Ulla Schultheiß, Fruzsina Kotsis, Simone Meder, Erna Mitsch, Ursula Reinhard, Gerd Walz; RWTH Aachen University: Jürgen Floege, Georg Schlieper, Turgay Saritas, Sabine Ernst, Nicole Beaujean; Charité, University Medicine Berlin: Elke Schaeffner, Seema Baid-Agrawal, Kerstin Theisen; Hannover Medical School: Hermann Haller, Jan Menne; University of Heidelberg: Martin Zeier, Claudia Sommerer, Rebecca Woitke; University of Jena: Gunter Wolf, Martin Busch, Rainer Fuß; Ludwig-Maximilians University of München: Thomas Sitter, Claudia Blank; University of Würzburg: Christoph Wanner, Vera Krane, Antje Börner-Klein, Britta Bauer; Medical University of Innsbruck, Division of Genetic Epidemiology: Florian Kronenberg, Julia Raschenberger, Barbara Kollerits, Lukas Forer, Sebastian Schönherr, Hansi Weissensteiner; University of Regensburg, Institute of Functional Genomics: Peter Oefner, Wolfram Gronwald, Helena Zacharias; Department of Medical Biometry, Informatics and Epidemiology (IMBIE),

University Hospital of Bonn: Matthias Schmid, Jennifer Nadal. The full list can be found in at http://www.gckd.org.

The Polish Kidney Genetics (POLYGENES) Network:

We thank the POLYGENES network for referring membranous nephropathy patients across Poland for this study. The POLYGENES network represents a collaborative group of Polish nephrologists, pediatricians, and geneticists that aims to advance genetics studies of Polish patients affected by inherited forms of kidney disease. The network is coordinated by the Polish Registry of Congenital Malformations (PRCM) in the Department of Genetics at Poznań University of Medical Sciences and includes a large number of clinical field centers across Poland. The key members include: Anna Latos-Bieleńska Anna Materna-Kiryluk, Anna Jamry-Dziurla, Marcin Zaniew, Marcin Kołbuc, Krzysztof Pawlaczyk, Marek Karczewski, Michał Stronka, Lucyna Cichanska, Zofia Adamska, Krzysztof Mucha, Leszek Pączek, Agnieszka Perkowska-Ptasińska, Ewa Komuda-Leszek, Małgorzata Mizerska-Wasiak, Grażyna Krzemień, Agnieszka Szmigielska, Ryszard Grenda, Dariusz Runowski, Ireneusz Habura, Dorota Drożdż,Monika Miklaszewska, Katarzyna Zachwieja, Przemysław Sikora, Beata Bieniaś, Marcin Tkaczyk, Daria Tomczyk, Anna Krakowska, Katarzyna Siniewicz-Luzeńczyk,Anna Jander, Tomasz Jarmoliński, Norbert Kwella, Klaudia Korecka, Maria Szczepanska, Piotr Adamczyk, Edyta Machura, Tomasz Hryszko, Bolesław Rutkowski, Izabella Kuźmiuk-Glembin, Ewa Król, Barbara Bullo-Piontecka, Andrzej Brodkiewicz. The full list of collaborating sites and clinicians can be found at http://www.polygenes.org.

The Population Architecture Using Genomics and Epidemiology (PAGE) Consortium:

We would like to thank the PAGE consortium for providing over 50,000 of MEGA chip-genotyped multiethnic population controls for the purpose of this study. The PAGE consortium is funded by the National Human Genome Research Institute (NHGRI) with co-funding from the National Institute on Minority Health and Health Disparities (NIMHD). The PAGE Coordinating Center (U01HG007419) assisted with genotype data management, analysis pipelines, and general study coordination. Genotyping services were provided by the Center for Inherited Disease Research (CIDR). CIDR is fully funded through a federal contract from the National Institutes of Health to The Johns Hopkins University, contract number HHSN268201200008I. Genotype data quality control and quality assurance services were provided by the Genetic Analysis Center in the Biostatistics Department of the University of Washington, through support provided by the CIDR contract. The control data included in this manuscript result from collaboration between the following studies and organizations: the Bio*Me* Biobank (U01HG007417) in The Charles Bronfman Institute for Personalized Medicine at the Icahn School of Medicine at Mount Sinai, in New York; the Hispanic Community Health Study/Study of Latinos (U01HG007416); the multi-site Multiethnic Cohort study (U01HG007397); and the multi-site Women's Health Initiative (U01HG007376).

The Nephrotic Syndrome Study Network (NEPTUNE):

The NEPTUNE Study Enrolling Centers Include: Case Western Reserve University, Cleveland, OH (J Sedor[*], K Dell[**], M Schachere[#], J Negrey); Children's Hospital, Los Angeles, CA (K Lemley[*], S Tang[#]); Children's Mercy Hospital, Kansas City, MO (T Srivastava[*], A Garrett[#]); Cohen Children's Hospital, New Hyde Park, NY (C Sethna[*], K Grammatikopoulos[#], R Odusayana); Columbia University, New York, NY (G Appel[*], M Toledo[#]); Emory University, Atlanta, GA (L Greenbaum[*], C Wang[**], B Lee[#]); Harbor-University of California Los Angeles Medical Center (S Adler[*], C Nast[*‡], J LaPage[#]); John H. Stroger Jr. Hospital of Cook County, Chicago, IL (A Athavale[*]); Johns Hopkins Medicine, Baltimore, MD (A Neu[*], S Boynton[#]); Mayo Clinic, Rochester, MN (F Fervenza[*], M Hogan[**], J Lieske[*], V Chernitskiy[#]); Montefiore Medical Center, Bronx, NY (F Kaskel[*], N Kumar[*], P Flynn[#]); NIDDK Intramural, Bethesda MD (J Kopp[*], J Blake[#]); New York University Medical Center, New York, NY (H Trachtman[*], O Zhdanova[**], F Modersitzki[#], S Vento[#]); Stanford University, Stanford, CA (R Lafayette[*], K Mehta[#]); Temple University, Philadelphia, PA (C Gadegbeku[*], D Johnstone[**], S Quinn-Boyle); University Health Network Toronto (D Cattran[*], M Hladunewich[**], H Reich[**], P Ling[#], M Romano[#]); University of Miami, Miami, FL (A Fornoni[*], L Barisoni[*], C Bidot[#]); University of Michigan, Ann Arbor, MI (M Kretzler[*], D Gipson[*], A Williams[#], R Pitter[#]); University of North Carolina, Chapel Hill, NC (V Derebail[*], K Gibson[*], S Grubbs[#], A Froment[#]); University of Pennsylvania, Philadelphia, PA (L Holzman[*], K Meyers[**], K Kallem[#], A Swensen[#]); University of Texas Southwestern, Dallas, TX (K Sambandam[*], E Brown[**], Z Wang[#]); University of Washington, Seattle, WA (A Jefferson[*], S Hingorani[**], K Tuttle[**§], L Curtin[#], S Dismuke[#], A Cooper[#§]); Wake Forest University, Winston-Salem, NC (B Freedman[*], JJ Lin[**], S Gray[#]); Data Analysis and Coordinating Center (M Kretzler, L Barisoni, C Gadegbeku, B Gillespie, D Gipson, L Holzman, L Mariani, M Sampson, P Song, J Troost, J Zee, E Herreshoff, S Li, C Lienczewski, T Mainieri, M Wladkowski, A Williams, D Zinsser); National Institute of Diabetes and Digestive and Kidney Diseases (NIDDK) Program Office (K Abbott, C Roy); The National Center for Advancing Translational Sciences (NCATS) Program Office (T Urv, PJ Brooks).

[*]Principal Investigator; [**]Co-investigator, [#]Study Coordinator
[‡]Cedars-Sinai Medical Center, Los Angeles, CA
[§]Providence Medical Research Center, Spokane, WA

SUPPLEMENTARY METHODS:

The following case-control cohorts were included in the GWAS analyses:

Chinese Discovery Cohort:

The cases and controls were recruited from the clinics of the Department of Nephrology of Ruijin Hospital, Shanghai Jiao Tong University School of Medicine, Shanghai, China. All cases had a biopsy-established diagnosis of idiopathic MN and provided informed consent to participate in genetic studies. Any cases of secondary MN due to drugs, malignancy, infection, or autoimmune disease were excluded. Genomic DNA was extracted from whole blood samples using standard procedures and genotyping was performed with Infinium OmniZhonghua-8 Beadchip. The genotyping was performed by Genergy Bio (Shanghai, China). The analysis of intensity clusters and genotype calls were performed in Illumina Genome Studio software. All SNPs were called on forward DNA strand. The standard quality control (QC) included per-SNP genotyping rate >95%, per-individual genotyping rate >90%, MAF >0.01, and HWE test p-value >$1x10^{-5}$ in controls. The duplicates and cryptic relatedness in the given cohort were determined and excluded based on the estimated pairwise kinship coefficients >0.05. Gender of each individual was imputed based on the analysis of sex chromosome markers and cases with mismatched gender were excluded. The ancestry was evaluated using principal component analyses (PCA)[3] using 30,406 high quality (genotype rate > 99%), common (MAF>1%), and independent (pairwise r2 < 0.05) markers. The regions of high LD were excluded from this analysis (see below). Based on the distribution of individuals along PC axes, ancestry outliers were excluded. The final PCA produced three significant PCs by Tracy-Widom test and demonstrated successful matching of cases and controls by population ancestry (**Supplementary Figure 1a**). After QC, the final dataset comprised of 1,465 individuals (561 cases and 904 controls) genotyped with 865,296 markers with an overall genotyping rate 99.8%. We carried out imputation analysis using East Asian reference populations from Phase 3 1000 Genomes Projects using Minimac3[4] with pre-phasing in MACH 1.0 software. A total of 6,794,947 high quality common markers ($R^2 > 0.8$ and $MAF > 0.01$) were imputed and used in downstream analyses.

South Korean Discovery Cohort:

The cases and controls were recruited from Seoul National University Hospital, Seoul, South Korea. A total of 166 cases were diagnosed with idiopathic MN by percutaneous kidney biopsy performed in Seoul National University Hospital between years 2009 and 2015. Any cases of secondary MN due to malignancy, infection, autoimmune disease, or drugs were excluded. From the same institution, 979 control samples were recruited among subjects without evidence of chronic kidney disease, urinary abnormalities, diabetes, hypertension or cancer. All participants provided informed consent to participate in genetic studies. DNA of whole blood samples was extracted using standard procedures and genotyping was performed using the Illumina Multiethnic Global Ancestry (MEGA) chip. The analysis of intensity clusters and genotype calls were performed in Illumina Genome Studio software; all SNPs were called on forward DNA strand and standard QC filters were applied, including per-SNP genotyping rate >95%, per-

individual genotyping rate >90%, MAF >0.01, and HWE test p-value $>1 \times 10^{-5}$ in controls. Additionally, the duplicates/cryptic relatedness, gender mismatches and ancestry outliers were excluded using same thresholds as above. The final PCA using 34,199 high-quality independent markers demonstrated excellent genetic matching between cases and controls, resulting in one significant PC of ancestry by Tracy-Widom test (**Supplementary Figure 1b**). The regions of high LD were excluded from the PCA analysis (see below). After QC, the dataset consisted of 872 individuals (164 cases and 708 controls) genotyped for 613,185 SNPs with overall genotyping rate of 99.9%. The imputation analysis was performed using MACH 1.0 for pre-phasing and then Minimac3 for imputation based on the 1000 Genomes (Phase 3) East Asian reference. A total of 6,237,121 common markers imputed at high quality ($R^2 > 0.8$ and $MAF > 0.01$) were used in downstream analyses.

Japanese Discovery Cohort:

The cases and controls were recruited from the Division of Nephrology, Juntendo University Faculty of Medicine in Tokyo, Japan. All subjects provided informed consent to participate in genetic studies. The case status was defined by a kidney biopsy diagnosis of idiopathic MN. Any cases of secondary MN due to malignancy, infection, autoimmune disease, or drugs were excluded. The genotyping was performed using Illumina MEGA chip; the analysis of intensity clusters and genotype calls were performed in Illumina Genome Studio software with all SNPs called on forward DNA strand. The quality control filters were identical to the ones used in the analysis of Chinese and South Korean cohorts above. After QC, 439 individuals (81 cases and 358 controls) and 624,535 markers with mean genotyping rate 99.9% were retained in the analysis. For the purpose of PCA, a subset of 30,609 high-quality independent (r2 < 0.05) SNPs were selected. The regions of high LD were excluded from the PCA analysis (see below). The PCA of the final dataset showed one significant PC by Tracy-Widom test, no outliers, and excellent matching between cases and controls (**Supplementary Figure 1c**). Imputation was performed using East Asian reference from 1000 Genome Projects (Phase 3) with MACH 1.0 and Minimac3 used for phasing and imputation. A total of 6,309,855 common markers imputed at high quality ($R^2 > 0.8$ and $MAF > 0.01$) were used in downstream analyses.

European Discovery Cohort 1:

The cases and controls were referred from several European medical centers and existing studies. This includes cases recruited from a large physician referral network in Italy and Poland, and individuals recruited in North America but self-identified as White/Europeans, such as cases recruited at the Glomerular Centers at Columbia University (New York, USA). All cases were diagnosed with idiopathic MN based on renal biopsy and provided informed consent to participate in genetic studies. Suspected cases of secondary MN were excluded. DNA of whole blood samples was extracted using standard procedures and genotyped using Illumina MEGA chip. The analysis of intensity clusters and genotype calls were performed using Illumina Genome Studio; all SNPs were called on forward DNA strand. Standard QC filters included per-SNP genotyping rate >95%, per-individual genotyping rate > 90%, MAF > 0.01, and HWE test

p-value > 1×10^{-5} in controls. The duplicates and cryptic relatedness among cases and controls were identified and excluded based on the estimated pairwise kinship coefficients >0.05. For PCA, we used 37,611 high-quality independent (pairwise r2 < 0.05) SNPs. The regions of high LD were excluded from the PCA analysis (see below). After exclusions of ancestry outliers, the PCA of the final dataset produced 5 significant PCs by Tracy-Widom test and visual inspection of PCA plots demonstrated adequate genetic-matching between cases and controls (**Supplementary Figure 1d**). The final dataset post-QC consisted of 1,857 individuals (611 cases and 1,246 controls) genotyped for 566,231 markers with an average genotyping rate of 99.8%. The imputation analysis was then carried out with Minimac3 after pre-phasing in Eagle V2.3[4,5] and using 1000 Genomes (Phase 3) European population reference. A total of 7,629,834 common high quality markers ($R^2 > 0.8$ and $MAF > 0.01$) were imputed and used in downstream analyses.

European Discovery Cohort 2:

A total of 1,284 biopsy-diagnosed cases of idiopathic were recruited in the UK and Germany. The UK cohort was collected by the UK MRC AUTO-MN Network (for the full list of participating centers, please see Supplementary Acknowledgments) and represents a mix of incident and prevalent cases of biopsy-proven primary MN diagnosed between 2013 and 2017. In addition, samples from historical cases of biopsy-proven MN collected from 1990 to 2017 from Central Manchester University Hospital and University Hospital of South Manchester (now united as Manchester University Hospital Foundation Trust) were also included. Additional cases were recruited by the Department of Nephrology at the University College London, London, UK, and the University Medical Center Hamburg-Eppendorf, Hamburg, Germany. Cases of secondary MN were excluded. All subjects provided informed consent to participate in genetic studies. DNA was extracted from whole blood based on the standard procedures and genotyping was performed with Illumina MEGA chip platform; analysis of intensity clusters and genotype calls were performed using the Illumina Genome Studio software. All SNPs were called on the forward DNA strand. After standard quality control filtering, 1,281 cases genotyped for 684,831markers were retained with the average genotyping rate of 99.7%. We performed ancestry-based matching of these cases with 49,401 available population controls (Columbia University CKD Biobank and the PAGE consortium) genotyped on the same platform (Illumina MEGA chip) and subjected to identical QC filters as the cases. After merging, and retaining overlapping markers between the datasets, ancestry matching was performed using iterative PCA with exclusions of case outliers and unmatchable controls. The PCA was based on 35,134 high-quality, common (MAF>0.01) independent (pairwise r2<0.05) markers. The regions of high LD were excluded from the PCA analysis (see below). After several rounds of case and control exclusions, the dataset was reduced to 2,216 individuals (1,071 cases and 1,145 ancestry-matched controls). The analysis of duplicates and cryptic relatedness was then carried out, and a total of 26 cases and 51 controls were excluded based on estimated pairwise kinship coefficients > 0.05, resulting in the final number of 2,139 individuals (1,045 cases and 1,094 controls). This dataset was re-subjected to final QC filters including per-SNP genotyping rate >95%, per-individual genotyping rate > 90%, MAF > 0.01, and HWE test p-value > 1×10^{-5} in controls. In

total, 621,778 markers and all 2,139 individuals passed all the filters with overall genotyping rate of 99.8%. The PCA of the final dataset resulted in 5 significant PCs by Tracy-Widom test and demonstrated successful ancestry matching between cases and controls (**Supplementary Figure 1e**). The imputation analysis was then carried out using European reference from 1000 Genome Project (Phase 3) with Minimac3/Eagle V2.3 pipeline as above[4,5]. A total of 7,638,044 common high quality imputed markers ($R^2 > 0.8$ and $MAF > 0.01$) were used in downstream analyses.

Turkish Discovery Cohort:

This cohort is composed of 680 individuals (288 cases and 392 controls) recruited from the following clinical nephrology centers and clinics in Turkey: Istanbul Faculty of Medicine, Istanbul University, Istanbul; Haseki Training and Research Hospital, Istanbul; Uludag University Faculty of Medicine, Bursa; Marmara University School of Medicine, Istanbul; and Sisli Hamidiye Etfal Training and Research Hospital, Istanbul. All cases had a biopsy-confirmed diagnosis of idiopathic MN and provided informed consent to participate in genetic studies. Any cases suspected to have a secondary form of MN were excluded. DNA of whole blood samples was extracted using standard procedures; genotyping was conducted using Illumina MEGA chip. The analysis of intensity clusters and genotype calls were performed in Illumina Genome Studio software and all SNPs were called on forward DNA strand. In total, 642 individuals (264 cases and 378 controls) and 708,556 overlapping markers passed our standard QC filters. This dataset was then subjected to duplicates and cryptic relatedness analyses. A total of 52 individuals (10 cases and 42 controls) were excluded based on pairwise kinship coefficients >0.05, resulting in the final number of 590 individuals (254 cases and 336 controls). The average genotyping rate of the final dataset was 99.8%. The PCA produced 5 significant PCs by Tracy-Widom test, demonstrated no ancestry outliers, and excellent ancestry matching between cases and controls (**Supplementary Figure 1f**). The imputation analysis was conducted with Minimac3 after phasing with Eagle v.2.3 using Phase 3 1000 Genome Project European populations as reference. A total of 7,388,236 common high quality markers ($R^2 > 0.8$ and $MAF > 0.01$) were imputed using this strategy.

Sardinia Discovery Cohort:

The dataset consisted of 93 biopsy-confirmed primary (idiopathic) membranous nephropathy cases enrolled in Nephrology, Dialysis, and Transplantation Unit, Giuseppe Brotzu Hospital in Cagliari and 1,602 controls recruited in the Sardinian blood transfusion centers as previously described[6]. All individuals included in this cohort were of Sardinian ancestry and all provided a written consent to participate in genetic studies. DNA was extracted from whole blood samples and typed with two Illumina Infinium arrays: high-density OmniExpress and low-density targeted Immunochip array. Genotypes were called using Genome Studio. We applied standard QC filters including per-SNP genotyping rate >95%, per-individual genotyping rate > 90%, MAF > 0.01, and HWE test p-value > 1×10^{-5} in controls. The genotypes from the two arrays were then merged in a combined genetic map. Altogether, 591,922 common (MAF>0.01) autosomal markers were genotyped across the study samples. We tested for and removed

duplicate or identical twin samples. The final dataset of this cohort contained 1,591 individuals including 93 cases and 1,498 controls. Population substructure was characterized with PCA using a set of 27,553 independent autosomal markers. The PCA of final dataset showed 2 significant PCs by Tracy-Widom test and successful ancestry mapping between cases and controls (**Supplementary Figure 1g**). Genotypes were phased with SHAPEIT[7] and Minimac3[4] was used to impute markers using 1000 Genomes Phase 3 European reference population. The final analysis included 8,533,061 common markers imputed at high quality. Association testing was performed under additive model with adjustment for 2 significant PCs of ancestry using the test "b.firth" as implemented in EPACTS[8].

GCKD Discovery Cohort:

The GCKD study is a prospective cohort of 5,217 adult patients of European ancestry with moderate CKD at baseline recruited between years 2010 and 2012; the inclusion criteria involved either an estimated glomerular filtration rate (eGFR) between 30 and 60 mL/min/1.73 m^2 or overt proteinuria upon study inclusion. The patient characteristics from the baseline visit have been published previously[9]. We identified idiopathic MN as the leading cause of CKD in 151 of 5,217 GCKD patients, 147 of whom had a kidney biopsy confirmation of the diagnosis. We selected 1,655 ancestry-matched GCKD patients for whom CKD etiology was clearly assigned to a non-MN cause, including nephrosclerosis, infections, tumor nephrectomies, interstitial nephritis and vascular diseases, as previously described[10]. Genome-wide genotyping was performed using the Illumina Omni2.5Exome array; detailed QC of the genotype data has been described previously[11]. In total, 1,392,377 markers passed all QC filters. The PCA of this dataset produced 3 significant PCs by Tracy-Widom test and showed no ancestry outliers (**Supplementary Figure 1h**). Imputation was performed using the HRC 1.1 haplotype reference panel and 7,494,864 common (MAF>0.01) markers of high imputation quality ($R_2 > 0.8$) were retained in the analysis. Association analyses were performed for the final dataset using SNPTEST version 2.5.2.

Chinese Replication Cohort:

This cohort consisted of 618 cases and 849 controls recruited by the Renal Division in the Department of Medicine of Peking University First Hospital, and additional 238 cases and 400 controls recruited at the Department of Nephrology of Shanghai Ruijin Hospital and Xin Hua Hospital, both hospitals affiliated with Shanghai Jiao Tong University School of Medicine, Shanghai, China. All cases and controls were of self-reported Han Chinese ethnicity. All participants provided an informed consent to participate in genetic studies. All cases had a biopsy-confirmed diagnosis of idiopathic MN; secondary causes of MN were excluded. Genomic DNA was extracted from whole blood using standard methods. The genotyping was performed using Kompetitive Allele Specific PCR (KASP), a proprietary genotyping technology developed by LGC Genomics. The genotyping was performed by LGC Genomics (Shanghai, China). The genotype calls were determined using an automated clustering algorithm implemented in SNP Viewer version 1.99 (KBiosciences, 2008). The genotype clusters were also examined visually

across all plates to assure lack of technical artifacts. The overall genotyping rate across all samples was 99%. For quality control we calculated minor allele frequencies, as well as per-SNP and per-individual rates of missingness. Additionally, we tested for Hardy-Weinberg equilibrium among the controls to assure lack of bias due to genotyping artifacts or population stratification. Any individual sample with more than 10% missing genotypes was excluded from the analysis. All SNPs in the final analyses had missingness <5% and all passed HWE test in controls (P>0.01). After implementation of all quality control filters, 826 cases and 1,239 controls were retained in the final analysis.

The UK validation cohort:

This cohort was composed of a total of 684 individuals (335 cases and 349 controls) collected by the MRC/Kidney Research UK National DNA Bank for Glomerulonephritis. Serum testing for anti-PLA2R antibody was performed using the EUROIMMUN ELISA as described below; 176 cases with complete genotype data serum tested within 6 months of kidney biopsy were used for validations of combined serum-genetic tests. The cases were genotyped using the HumanCNV370-Quad SNP chip (Illumina). The ethnically-matched controls were recruited as part of the 1958 UK Birth Control study and were genotyped using the HumanHap300 SNP chip (Illumina). All genotyping was performed by deCODE Genetics, Iceland. The joint imputation was performed after stringent genotype quality control as described previously[12]. The GRS was successfully calculated for 333 cases and 348 controls.

The French validation cohort:

This cohort was composed of a total of 232 individuals (75 cases and 157 controls) recruited by the GNProgress Study. In total, there were 72 cases with serum tested for anti-PLA2R antibody at the time of biopsy. Serum testing was performed using the IIFT method (IIFT Mosaic; EUROIMMUN AG, Lübeck, Germany) according to manufacturer's instruction. Negativity of anti-PLA2R was defined as absence of detectable antibodies at serum dilution of 1/10. Antibody positivity was defined as positive staining at serum dilutions of 1/10 or higher. In anti-PLA2R-positive patients, quantitative measurements were performed using different serum dilutions (1/10, 1/100, and 1/1000). Genotyping of all cases and controls was performed using the HumanCNV370-Duo SNP chip (Illumina) by the Centre National de Génotypage, Evry, France. The imputation was performed after stringent genotype quality control as described previously[12]. After imputation, the GRS was calculated for all 75 cases and 157 controls.

The Dutch validation cohort:

This cohort was composed of a total of 1,978 individuals (146 cases and 1,832 controls). Serum measurements were available for 146 cases with genetic data. Serum testing for anti-PLA2R antibody was performed using the EUROIMMUN ELISA as described below; 72 cases with complete genotype data and serum tested within 6 months of kidney biopsy were used for validations of combined serum-genetic tests. The cases were genotyped using HumanCNV370-

Quad SNP chip (Illumina) by University College London Genomics at the Institute of Child Health, London. The controls were recruited by the Nijmegen Biomedical Study and were genotyped with HumanCNV370-Duo SNP chip (Illumina) by deCODE Genetics, Reykjavik, Iceland. The imputation was performed after stringent genotype quality control as described previously[12]. After imputation, the GRS was successfully calculated for 142 cases and 1,832 controls.

The European serum control cohort:

We assembled an independent cohort of healthy controls was composed of a total of 145 unrelated individuals of European ancestry without any kidney disease, recruited as volunteers at Columbia University. Serum anti-PLA2R antibody titers in all individuals were determined using the EUROIMMUN ELISA as per manufacturer's protocol. All individuals were genotyped with the MEGA chip (Illumina) at the University of Michigan genotyping facility. The standard genotype quality control included the following filters: per individual and per SNP genotyping rates >95%, MAF >1%, and P-value for HWE test in controls $>1 \times 10^{-4}$. We performed genotype-based sex and relatedness checks, and detected no sex discordances against biobank records and no cryptically related individuals. The European ancestry was confirmed by PCA analysis with the European reference populations of the 1000 Genomes (Phase 3) Project. After the quality control analysis, the imputation was performed and the GRS was calculated for each individual as described above.

The NEPTUNE validation cohort:

The Nephrotic Syndrome Study Network (NEPTUNE) is a prospective, longitudinal cohort recruiting participants with substantial proteinuria at the time of first kidney biopsy[13]. The recruitment took place across 23 clinical centers in the U.S. and Canada, and involved individuals of diverse ancestries. Phenotypic data, urine, and blood samples were collected at baseline and over time. The tissue diagnosis was determined by a centralized review of kidney biopsy slides by the NEPTUNE pathology committee. In addition to MN, FSGS, and MCD, a smaller group of patients with IgA Nephropathy (IgAN) were also enrolled in the study. All NEPTUNE participants underwent low-depth whole genome sequencing (Illumina Hi-Seq), which takes advantage of shared haplotypes to make accurate genotype calls[14]; GotCloud was used for alignment and variant calling[15]. A subset of patients also underwent Illumina Exome Chip genotyping (Illumina); using chip data as a gold standard, we calculated WGS variant call concordance at 77,769 shared non-monomorphic sites; 97.5% of sites had > 97% concordance. Additional QC analyses were performed as previously described[16]. The PCA analysis was used to assign genetic ancestry to all NEPTUNE participants based on co-clustering with reference populations of the 1000 Genomes Phase 3. All top SNPs from our discovery GWAS were called in the WGS data with high confidence. Using the European discovery equation, the GRS was successfully determined for N=475 NEPTUNE participants. This included 89 cases with the central pathology diagnosis of primary MN and 386 disease controls, including 184 with FSGS, 164 with MCD, and 38 with IgAN. Serum levels of anti-PLA2R antibodies were determined using EUROIMMUN ELISA in 390 participants with available serum at the time of biopsy. Of

319 disease controls, one case of European ancestry with MCD tested positive with borderline PLA2R antibody level of 22.5 U/mL. Follow-up review of the renal biopsy revealed no histologic features of MN and no sub-epithelial deposits by electron microscopy; this individual was retained as a disease control in the analysis.

Regions of extended LD excluded from PCA-based ancestry analyses:

The following segments of high LD were excluded from the analyses of genetic ancestry by PCA as previously recommended[17]. The columns include chromosome, segment start position, and segment stop position; genomic position coordinates provided in bps (hg19).

```
1 48227413 52227412
2 86000000 100500000
2 183291755 190291755
3 47524996 50024996
3 83417310 86917310
5 44500000 50500000
5 128972101 131972101
6 25392021 33392022
6 57000000 64000000
6 139958307 142458307
7 55000000 66000000
8 7962590 11962591
8 43000000 50000000
8 111930824 114930824
10 37000000 43000000
11 87860352 90860352
12 33000000 40000000
20 32536339 35066586
```

SUPPLEMENTARY REFERENCES:

1. Lake BB, Chen S, Hoshi M, et al. A single-nucleus RNA-sequencing pipeline to decipher the molecular anatomy and pathophysiology of human kidneys. Nat Commun 2019;10:2832.
2. Bobart SA, De Vriese AS, Pawar AS, et al. Noninvasive diagnosis of primary membranous nephropathy using phospholipase A2 receptor antibodies. Kidney Int 2019;95:429-38.
3. Patterson N, Price AL, Reich D. Population structure and eigenanalysis. PLoS Genet 2006;2:e190.
4. Das S, Forer L, Schonherr S, et al. Next-generation genotype imputation service and methods. Nat Genet 2016;48:1284-7.
5. Loh PR, Danecek P, Palamara PF, et al. Reference-based phasing using the Haplotype Reference Consortium panel. Nature Genetics 2016;48:1443-8.
6. Zoledziewska M, Costa G, Pitzalis M, et al. Variation within the CLEC16A gene shows consistent disease association with both multiple sclerosis and type 1 diabetes in Sardinia. Genes Immun 2009;10:15-7.
7. Delaneau O, Marchini J, Zagury JF. A linear complexity phasing method for thousands of genomes. Nat Methods 2011;9:179-81.
8. Kang HM, Sul JH, Service SK, et al. Variance component model to account for sample structure in genome-wide association studies. Nat Genet 2010;42:348-54.
9. Titze S, Schmid M, Kottgen A, et al. Disease burden and risk profile in referred patients with moderate chronic kidney disease: composition of the German Chronic Kidney Disease (GCKD) cohort. Nephrol Dial Transplant 2015;30:441-51.
10. Wunnenburger S, Schultheiss UT, Walz G, et al. Associations between genetic risk variants for kidney diseases and kidney disease etiology. Sci Rep 2017;7:13944.
11. Li Y, Sekula P, Wuttke M, et al. Genome-Wide Association Studies of Metabolites in Patients with CKD Identify Multiple Loci and Illuminate Tubular Transport Mechanisms. J Am Soc Nephrol 2018;29:1513-24.
12. Stanescu HC, Arcos-Burgos M, Medlar A, et al. Risk HLA-DQA1 and PLA(2)R1 alleles in idiopathic membranous nephropathy. The New England journal of medicine 2011;364:616-26.
13. Gadegbeku CA, Gipson DS, Holzman LB, et al. Design of the Nephrotic Syndrome Study Network (NEPTUNE) to evaluate primary glomerular nephropathy by a multidisciplinary approach. Kidney Int 2013;83:749-56.
14. Li Y, Sidore C, Kang HM, Boehnke M, Abecasis GR. Low-coverage sequencing: implications for design of complex trait association studies. Genome Res 2011;21:940-51.
15. Jun G, Wing MK, Abecasis GR, Kang HM. An efficient and scalable analysis framework for variant extraction and refinement from population-scale DNA sequence data. Genome Res 2015;25:918-25.
16. Gillies CE, Putler R, Menon R, et al. An eQTL Landscape of Kidney Tissue in Human Nephrotic Syndrome. Am J Hum Genet 2018;103:232-44.
17. Anderson CA, Pettersson FH, Clarke GM, Cardon LR, Morris AP, Zondervan KT. Data quality control in genetic case-control association studies. Nat Protoc 2010;5:1564-73.
