# Supplementary material for: The genetic architecture of membranous nephropathy and its potential to improve non-invasive diagnosis
Source: Nat Commun. 2020 Mar 30;11:1600. doi: 10.1038/s41467-020-15383-w (PMC7105485; doi:10.1038/s41467-020-15383-w)
Supplement: Supplementary file 3 — Reporting Summary [file 41467_2020_15383_MOESM3_ESM.pdf]

nature research

Corresponding author(s): Krzysztof Kiryluk

Last updated by author(s): 2/18/2020

Reporting Summary

Nature Research wishes to improve the reproducibility of the work that we publish. This form provides structure for consistency and transparency in reporting. For further information on Nature Research policies, see Authors & Referees and the Editorial Policy Checklist .

Statistics

For all statistical analyses, confirm that the following items are present in the figure legend, table legend, main text, or Methods section.

n/a	Confirmed	
☐	☒	The exact sample size (*n*) for each experimental group/condition, given as a discrete number and unit of measurement
☐	☒	A statement on whether measurements were taken from distinct samples or whether the same sample was measured repeatedly
☐	☒	The statistical test(s) used AND whether they are one- or two-sided<br>*Only common tests should be described solely by name; describe more complex techniques in the Methods section.*
☐	☒	A description of all covariates tested
☐	☒	A description of any assumptions or corrections, such as tests of normality and adjustment for multiple comparisons
☐	☒	A full description of the statistical parameters including central tendency (e.g. means) or other basic estimates (e.g. regression coefficient) AND variation (e.g. standard deviation) or associated estimates of uncertainty (e.g. confidence intervals)
☐	☒	For null hypothesis testing, the test statistic (e.g. *F*, *t*, *r*) with confidence intervals, effect sizes, degrees of freedom and *P* value noted<br>*Give P values as exact values whenever suitable.*
☒	☐	For Bayesian analysis, information on the choice of priors and Markov chain Monte Carlo settings
☒	☐	For hierarchical and complex designs, identification of the appropriate level for tests and full reporting of outcomes
☐	☒	Estimates of effect sizes (e.g. Cohen's *d*, Pearson's *r*), indicating how they were calculated

Our web collection on statistics for biologists contains articles on many of the points above.

Software and code

Policy information about availability of computer code

Data collection	NA
Data analysis	Software used: Illumina Genome Studio 2.0.4, PLINK 1.9, Minimac 3, Eagle 2.3, EIGENSOFT, EPACTS, SNPTEST v2.5.2, VCFtools, IGV 2.0.1, FUN-LDA, LDSC, and R v.3.4.8 (CRAN).

For manuscripts utilizing custom algorithms or software that are central to the research but not yet described in published literature, software must be made available to editors/reviewers. We strongly encourage code deposition in a community repository (e.g. GitHub). See the Nature Research guidelines for submitting code & software for further information.

Data

Policy information about availability of data

All manuscripts must include a data availability statement. This statement should provide the following information, where applicable:
- Accession codes, unique identifiers, or web links for publicly available datasets
- A list of figures that have associated raw data
- A description of any restrictions on data availability

All genome-wide summary statistics are freely available for download on our lab website: www.columbiamedicine.org/divisions/kiryluk/resources.php. The calculations of genetic risk score (GRS) and combined risk score (CRS) are implemented in the form of an online risk calculator, which is also freely available on our lab website. The PAGE consortium control genotype data is available on dbGAP under accession number phs000356.v2.p1. Primary genotype data for European-1 cohort will be available on dbGAP, accession number pending. Our IRB determined that the use of this dataset is restricted to genetic studies of kidney disease. Because of consent restrictions and/or country-specific privacy laws, we are unable to share primary genotype data on dbGAP for other international cohorts. All data and summary statistics are available from the corresponding authors upon reasonable request.

Field-specific reporting

Please select the one below that is the best fit for your research. If you are not sure, read the appropriate sections before making your selection.

- [x] Life sciences
- [ ] Behavioural & social sciences
- [ ] Ecological, evolutionary & environmental sciences

For a reference copy of the document with all sections, see nature.com/documents/nr-reporting-summary-flat.pdf

Life sciences study design

All studies must disclose on these points even when the disclosure is negative.

Sample size	The primary analysis was performed in 12,820 individuals (3,782 kidney biopsy-diagnosed cases and 9,038 controls) across 9 international cohorts as detailed in Table 1. Eight of the nine cohorts were genotyped genome-wide and the 9th cohort was genotyped by targeted SNP typing.
Data exclusions	Any cases with suspected secondary cause of MN, concurrent malignancy, or systemic lupus erythematosus were excluded.
Replication	We used the previously published European case-control cohorts for the purpose of GRS/CRS validation studies (referred to as the UK, the French, and the Dutch validation cohorts). In addition, we validated our findings in the NEPTUNE cohort of patients with incident nephrotic syndrome.
Randomization	NA
Blinding	NA

Reporting for specific materials, systems and methods

We require information from authors about some types of materials, experimental systems and methods used in many studies. Here, indicate whether each material, system or method listed is relevant to your study. If you are not sure if a list item applies to your research, read the appropriate section before selecting a response.

Materials & experimental systems

n/a	Involved in the study	
x		Antibodies
x		Eukaryotic cell lines
x		Palaeontology
x		Animals and other organisms
	x	Human research participants
x		Clinical data

Methods

n/a	Involved in the study	
x		ChIP-seq
x		Flow cytometry
x		MRI-based neuroimaging

Human research participants

Policy information about studies involving human research participants

Population characteristics	The detailed description of study cohorts is provided in the supplement. All cases were defined by a kidney biopsy diagnosis of primary MN and had the following characteristics:<br>Chinese discovery cohort: mean age 53; sex ratio (M:F) 1.4:1; East Asian.<br>Japanese discovery cohort: mean age 53; sex ratio (M:F) 1:1; East Asian.<br>Korean discovery cohort: no age available; sex ratio (M:F) 1.5:1; East Asian.<br>European discovery 1 cohort: mean age 53; sex ratio (M:F) 0.9:1; European.<br>European discovery 2 cohort: mean age 57; sex ratio (M:F) 1.2:1; European.<br>Turkish discovery cohort: age information not available; sex ratio (M:F) 1.2:1; European.<br>Sardinian discovery cohort: genome-wide summary statistics provided by the SardiNIA study.<br>GCKD discovery cohort: genome-wide summary statistics provided by the GCKD consortium.<br>Chinese replication cohort: age and gender not available; East Asian.<br>UK validation cohort: mean age 53; sex ratio (M:F) 2.2:1; European.<br>Dutch validation cohort: mean age 52; sex ratio (M:F) 2.9:1; European.<br>French validation cohort: mean age 50; sex ratio (M:F) 3.4:1; European.<br>NEPTUNE validation cohort: mean age 33; sex ratio (M:F) 1.4:1; Multi-ethnic North American-based cohort of patients with incident nephrotic syndrome.
Recruitment	Because primary membranous nephropathy (MN) represents a rare kidney phenotype, we aimed to recruit all cases of biopsy confirmed MN across all institutions/studies contributing to this work. This includes incident cases (recruited at the time of kidney biopsy) as well as prevalent cases (recruited anytime after their diagnostic biopsy), and individuals who received a kidney transplant because of end stage renal disease due to primary MN.
Ethics oversight	The study was approved by the Columbia University IRB under the IRB protocol number AAAC7385 as well as by the local ethics committees at all sites contributing biosamples and clinical data to this study.

Note that full information on the approval of the study protocol must also be provided in the manuscript.
